# Supplementary material for: Transcriptome Analysis of Fusarium Root-Rot-Resistant and -Susceptible Alfalfa (Medicago sativa L.) Plants during Plant–Pathogen Interactions
Source: Genes (Basel). 2022 Apr 28;13(5):788. doi: 10.3390/genes13050788 (PMC9140628; doi:10.3390/genes13050788)
Supplement: Supplementary file 1 [file genes-13-00788-s001.zip › Table S11.pdf]

Table S11: Expression difference of three CDPK genes in susceptible clone line at four time points between inoculated and uninoculated group.

<table>
<tr><th></th><th>24h</th><th></th><th></th><th></th><th>48h</th><th></th><th></th><th></th><th>72h</th><th></th><th></th><th></th><th>7d</th><th></th><th></th><th></th><th></th><th></th><th></th><th></th></tr>
<tr><th>Gene ID</th><th>log₂FoldChange</th><th>pval</th><th>padj</th><th>Regulation</th><th>log₂FoldChange</th><th>pval</th><th>padj</th><th>Regulation</th><th>log₂FoldChange</th><th>pval</th><th>padj</th><th>Regulation</th><th>log₂FoldChange</th><th>pval</th><th>padj</th><th>Regulation</th><th>IPR</th><th>TREMBL</th><th>SwissProt</th><th>NR</th></tr>
<tr><td>MS.gene41297</td><td>0.338008301</td><td>0.335627152</td><td>0.693731237</td><td>Nodiff</td><td>0.215997689</td><td>0.427430309</td><td>1</td><td>Nodiff</td><td>-1.342992105</td><td>2.48189E-07</td><td>4.68814E-06</td><td>Down Regulation</td><td>-1.41738452</td><td>3.93E-05</td><td>0.000493124</td><td>Down Regulation</td><td>IPR002048</td><td>tr|G7I2I2|G7I2I2_MEDTR</td><td>sp|Q06850|CDPK1_ARATH</td><td>XP_003589907.1</td></tr>
<tr><td>MS.gene50367</td><td>0.572324609</td><td>0.709769877</td><td>0.99161497</td><td>Nodiff</td><td>1.146641568</td><td>0.884042614</td><td>1</td><td>Nodiff</td><td>2.621308004</td><td>0.042214768</td><td>0.176380193</td><td>Nodiff</td><td>Inf</td><td>0.000552052</td><td>0.004908411</td><td>Up Regulation</td><td>IPR011009</td><td>tr|A0A072UFA6|A0A072UFA6_MEDTR</td><td>sp|P28582|CDPK_DAUCA</td><td>XP_013454393.1</td></tr>
<tr><td>MS.gene60795</td><td>2.003491341</td><td>0.01148052</td><td>0.055100198</td><td>Nodiff</td><td>0.1921603</td><td>0.578545085</td><td>1</td><td>Nodiff</td><td>1.329809966</td><td>0.000772307</td><td>0.006445847</td><td>Up Regulation</td><td>1.0671402</td><td>0.019959596</td><td>0.097297098</td><td>Nodiff</td><td>IPR011009</td><td>tr|A0A371F863|A0A371F863_MUCPR</td><td>sp|Q39016|CDPKB_ARATH</td><td>RDX74472.1</td></tr>
</table>
