# Supplementary material for: Transcriptome Analysis of Fusarium Root-Rot-Resistant and -Susceptible Alfalfa (Medicago sativa L.) Plants during Plant–Pathogen Interactions
Source: Genes (Basel). 2022 Apr 28;13(5):788. doi: 10.3390/genes13050788 (PMC9140628; doi:10.3390/genes13050788)
Supplement: Supplementary file 1 [file genes-13-00788-s001.zip › Table S5.pdf]

Table S5: The top 30 DEGs in the inoculated compared to uninoculated groups of the two lines with differential fold change at four time points.

Resistant clonal lines							
Time	Gene ID	Putative function	Nr ID[1]	CR vs TR		GO	KEGG
				log_2FC[2]	FDR[3]		
24 h	*MS.gene37740*	miraculin [Medicago truncatula]	XP_003620185.1	9.81	3.42E-04	GO:0003674;GO:0004857;GO:0004866;GO:0030234;GO:0030414;GO:0061134;GO:0061135;GO:0098772	-
24 h	*MS.gene57814*	miraculin [Medicago truncatula]	XP_003620185.1	8.93	1.35E-05	GO:0003674;GO:0004857;GO:0004866;GO:0030234;GO:0030414;GO:0061134;GO:0061135;GO:0098772	-
24 h	*MS.gene41336*	polygalacturonase inhibitor [Medicago truncatula]	XP_013467252.1	8.79	9.26E-60	-	-
24 h	*MS.gene34631*	polygalacturonase inhibitor [Medicago truncatula]	XP_013445647.1	8.69	4.51E-12	GO:0003674;GO:0005488;GO:0005515	-
24 h	*MS.gene57819*	miraculin [Medicago truncatula]	XP_003620175.1	8.4	8.82E-08	GO:0003674;GO:0004857;GO:0004866;GO:0030234;GO:0030414;GO:0061134;GO:0061135;GO:0098772	-
24 h	*MS.gene043769*	uncharacterized protein LOC112421360 [Medicago truncatula]	XP_024638646.1	8.26	9.13E-27	-	-
24 h	*MS.gene021793*	isoliquiritigenin 2'-O-methyltransferase isoform X1 [Medicago truncatula]	XP_003621323.1	8.24	2.51E-08	GO:0003674;GO:0003824;GO:0005488;GO:0005515;GO:0008168;GO:0008171;GO:0016740;GO:0016741;GO:0046983	-
24 h	*MS.gene37741*	miraculin [Medicago truncatula]	XP_003620185.1	8.19	4.69E-10	GO:0003674;GO:0004857;GO:0004866;GO:0030234;GO:0030414;GO:0061134;GO:0061135;GO:0098772	-
24 h	*MS.gene72359*	Expansin-like B1[Arabidopsis thaliana]	AFK45939.1	8.18	2.92E-27	GO:0005575;GO:0005576;GO:0110165	-
24 h	*MS.gene37737*	miraculin [Medicago truncatula]	XP_003620175.1	8.15	2.96E-03	GO:0003674;GO:0004857;GO:0004866;GO:0030234;GO:0030414;GO:0061134;GO:0061135;GO:0098772	-
24 h	*MS.gene41335*	polygalacturonase inhibitor [Medicago truncatula]	XP_013467253.1	8.04	1.84E-23	GO:0003674;GO:0005488;GO:0005515	-
24 h	*MS.gene43357*	polygalacturonase inhibitor [Medicago truncatula]	XP_013467252.1	8.02	3.42E-06	-	-
24 h	*MS.gene034636*	adenine nucleotide alpha hydrolase superfamily protein [Medicago truncatula]	KEH41788.1	7.93	1.87E-08	-	-
24 h	*MS.gene05228*	polygalacturonase inhibitor [Medicago truncatula]	XP_003620175.1	7.77	1.35E-20	GO:0003674;GO:0004857;GO:0004866;GO:0030234;GO:0030414;GO:0061134;GO:0061135;GO:0098772	-
24 h	*MS.gene069488*	miraculin [Medicago truncatula]	XP_003620185.1	7.59	5.93E-15	GO:0003674;GO:0004857;GO:0004866;GO:0030234;GO:0030414;GO:0061134;GO:0061135;GO:0098772	-
24 h	*MS.gene69943*	pathogenesis-related protein PR-4 [Medicago truncatula]	XP_013468976.1	7.56	7.67E-19	GO:0006950;GO:0006952;GO:0008150;GO:0009605;GO:0009607;GO:0009617;GO:0009620;GO:0042742;GO:0043207;GO:0050832;GO:0050896;GO:0051704;GO:0051707;GO:0098542	-
24 h	*MS.gene57815*	miraculin	XP_003620185.1	7.55	1.24E-52	GO:0003674;GO:0004857;GO:0004866;GO:0030234;GO	-

		[Medicago truncatula]				:0030414;GO:0061134;GO:0061135;GO:0098772	
24 h	*MS.gene069445*	miraculin [Medicago truncatula]	XP_003620175.1	7.55	2.74E-04	GO:0003674;GO:0004857;GO:0004866;GO:0030234;GO:0030414;GO:0061134;GO:0061135;GO:0098772	-
24 h	*MS.gene06026*	uncharacterized protein LOC25502128 [Medicago truncatula]	XP_013447134.1	7.39	2.11E-30	GO:0000166;GO:0003674;GO:0005488;GO:0005524;GO:0008144;GO:0017076;GO:0030554;GO:0032553;GO:0032555;GO:0032559;GO:0035639;GO:0036094;GO:0043167;GO:0043168;GO:0097159;GO:0097367;GO:1901265;GO:1901363	-
24 h	*MS.gene25817*	metal tolerance protein 10 [Medicago truncatula]	XP_003601380.1	-7.72	7.49E-11	GO:0003674;GO:0005215;GO:0005575;GO:0006810;GO:0006811;GO:0006812;GO:0008150;GO:0008324;GO:0015075;GO:0016021;GO:0022857;GO:0031224;GO:0051179;GO:0051234;GO:0055085;GO:0110165	-
24 h	*MS.gene08201*	arabinogalactan protein 14 [Medicago truncatula]	XP_003624630.2	-7.73	1.51E-22	-	-
24 h	*MS.gene005376*	enolase-phosphatase E1 [Medicago truncatula]	XP_003591256.1	-7.8	7.91E-46	-	-
24 h	*MS.gene041781*	probable ubiquitin-conjugating enzyme E2 C isoform X1 [Medicago truncatula]	XP_003591943.1	-7.82	1.82E-11	-	-
24 h	*MS.gene054580*	60S ribosomal protein L7-4 [Medicago truncatula]	XP_013454579.1	-7.99	8.08E-37	GO:0000463;GO:0000470;GO:0003674;GO:0003735;GO:0005198;GO:0005575;GO:0006139;GO:0006364;GO:0006396;GO:0006725;GO:0006807;GO:0008150;GO:0008152;GO:0009987;GO:0015934;GO:0016070;GO:0016072;GO:0022625;GO:0032991;GO:0034470;GO:0034641;GO:0034660;GO:0043170;GO:0044237;GO:0044238;GO:0044391;GO:0046483;GO:0071704;GO:0090304;GO:1901360;GO:1990904	-
24 h	*MS.gene03055*	uncharacterized protein LOC11422023 [Medicago truncatula]	XP_003594548.2	-8.25	4.86E-07	-	-
24 h	*MS.gene033242*	1-deoxy-D-xylulose 5-phosphate reductoisomerase, chloroplastic [Medicago truncatula]	XP_003608963.2	-8.27	1.61E-32	GO:0000166;GO:0003674;GO:0003824;GO:0005488;GO:0005515;GO:0006629;GO:0006720;GO:0008150;GO:0008152;GO:0008299;GO:0008610;GO:0009058;GO:0009987;GO:0016491;GO:0016614;GO:0016616;GO:0030604;GO:0036094;GO:0043167;GO:0043168;GO:0043169;GO:0044237;GO:0044238;GO:0044249;GO:0044255;GO:0046872;GO:0048037;GO:0050661;GO:0050662;GO:0055114;GO:0070402;GO:0071704;GO:0097159;GO:1901265;GO:1901363;GO:1901576	-
24 h	*MS.gene071837*	class-10 pathogenesis-related protein 1 [Medicago truncatula]	XP_003594829.1	-8.42	7.95E-36	GO:0003674;GO:0004857;GO:0004864;GO:0005488;GO:0006950;GO:0006952;GO:0007165;GO:0008150;GO:0008289;GO:0009738;GO:0009755;GO:0009987;GO:0010427;GO:0019208;GO:0019212;GO:0019840;GO:0019888;GO:0030234;GO:0031406;GO:0033293;GO:0036094;GO:0038023;GO:0042562;GO:0043167;GO:0043168;GO:0043177;GO:0043178;GO:0050789;GO:0050794;GO:0050896;GO:0060089;GO:0065007;GO:0098772	-
24 h	*MS.gene02575*	metal transporter Nramp5 [Medicago truncatula]	XP_003602055.1	-8.48	2.45E-03	GO:0003674;GO:0005215;GO:0005575;GO:0006810;GO:0006811;GO:0006812;GO:0008150;GO:0008324;GO:0015075;GO:0015318;GO:0016020;GO:0022857;GO:0022890;GO:0030001;GO:0046873;GO:0051179;GO:0051234;GO:0110165	-
24 h	*MS.gene25760*	heat shock 70 kDa protein 17 [Medicago truncatula]	XP_013461130.1	-9.63	1.6E-40	-	K09486
24 h	*MS.gene051009*	elongation factor	AET01475.1	-9.5	1.7E-71	GO:0000166;GO:0001882;GO:0001883;GO:0003674;GO	-

		1-alpha [Medicago truncatula]				:0003824;GO:0003924;GO:0005488;GO:0005525;GO:0016462;GO:0016787;GO:0016817;GO:0016818;GO:0017076;GO:0017111;GO:0019001;GO:0032549;GO:0032550;GO:0032553;GO:0032555;GO:0032561;GO:0035639;GO:0036094;GO:0043167;GO:0043168;GO:0097159;GO:0097367;GO:1901265;GO:1901363	
48 h	*MS.gene72002*	Expansin-like B1[Arabidopsis thaliana]	AFK45939.1	9.62	2.99E-02	GO:0005575;GO:0005576;GO:0110165	-
48 h	*MS.gene069488*	miraculin [Medicago truncatula]	XP_003620185.1	7.91	3.92E-02	GO:0003674;GO:0004857;GO:0004866;GO:0030234;GO:0030414;GO:0061134;GO:0061135;GO:0098772	-
48 h	*MS.gene51116*	uncharacterized protein LOC101494235 [Cicer arietinum]	XP_004491047.1	7.31	4.33E-02	-	-
48 h	*MS.gene071437*	putative ANTH domain-containing protein [Medicago truncatula]	RHN42370.1	6.91	1.16E-02	GO:0003674;GO:0005488;GO:0005515;GO:0005543;GO:0005545;GO:0005575;GO:0008150;GO:0008289;GO:0009987;GO:0016043;GO:0022607;GO:0030135;GO:0030136;GO:0030276;GO:0031410;GO:0031982;GO:0034622;GO:0035091;GO:0043167;GO:0043168;GO:0043226;GO:0043227;GO:0043229;GO:0043933;GO:0048268;GO:0065003;GO:0071840;GO:0097708;GO:0110165	-
48 h	*MS.gene43357*	polygalacturonase inhibitor [Medicago truncatula]	XP_013467252.1	6.36	2.99E-02	-	-
48 h	*MS.gene26988*	dihydroflavonol 4-reductase [Medicago sativa]	AEI59122.1	6.34	4.33E-02	GO:0003674;GO:0003824;GO:0005488;GO:0048037;GO:0050662	K13082
48 h	*MS.gene068813*	polygalacturonase inhibitor [Medicago truncatula]	XP_013445647.1	6.08	4.33E-02	-	-
48 h	*MS.gene015446*	protein NRT1/ PTR FAMILY 6.3 [Medicago truncatula]	XP_003611286.1	-4.75	3.92E-02	GO:0003674;GO:0005215;GO:0005575;GO:0006810;GO:0006857;GO:0008150;GO:0015833;GO:0016020;GO:0022857;GO:0042886;GO:0051179;GO:0051234;GO:0055085;GO:0071702;GO:0071705;GO:0110165	-
48 h	*MS.gene015460*	syntaxin-related protein KNOLLE [Medicago truncatula]	XP_003611259.1	-4.89	3.18E-02	GO:0003674;GO:0005484;GO:0005488;GO:0005515;GO:0005575;GO:0006810;GO:0006886;GO:0008104;GO:0008150;GO:0015031;GO:0015833;GO:0016020;GO:0016192;GO:0030674;GO:0033036;GO:0042886;GO:0045184;GO:0046907;GO:0051179;GO:0051234;GO:0051641;GO:0060090;GO:0071702;GO:0071705;GO:0110165	-
48 h	*MS.gene052898*	stilbene synthase 4 [Medicago truncatula]	XP_013461326.1	-4.89	2.70E-02	GO:0003674;GO:0003824;GO:0008150;GO:0008152;GO:0009058;GO:0016740;GO:0016746;GO:0016747	-
48 h	*MS.gene53436*	soyasaponin III rhamnosyltransferase [Cicer arietinum]	XP_004485550.1	-4.91	2.70E-02	GO:0003674;GO:0003824;GO:0016740;GO:0016757;GO:0016758	K18823
48 h	*MS.gene073769*	albumin-2 [Medicago truncatula]	XP_003590505.1	-4.94	3.39E-02	-	-
48 h	*MS.gene99438*	CAP, cysteine-rich secretory protein, antigen 5 [Medicago truncatula]	KEH31190.1	-4.95	3.97E-02	-	-
48 h	*MS.gene019669*	Ferric reduction oxidase 2[Arabidopsis thaliana]	XP_019053097.1	-5.02	4.08E-02	GO:0003674;GO:0003824;GO:0008150;GO:0008152;GO:0016491;GO:0055114	-
48 h	*MS.gene74528*	Putative stress up-regulated Nod	XP_003594548.2	-5.09	1.16E-02	-	-

		19[Medicago truncatula]					
48 h	*MS.gene055250*	protein SRG1-like [Medicago truncatula]	XP_013448902.1	-5.15	4.06E-02	GO:0003674;GO:0003824;GO:0008150;GO:0008152;GO:0016491;GO:0055114	-
48 h	*MS.gene84261*	Fe(III) reduction oxidase [Medicago truncatula]	AQQ72609.1	-5.23	4.06E-02	GO:0003674;GO:0003824;GO:0008150;GO:0008152;GO:0016491;GO:0055114	K00521
48 h	*MS.gene005844*	putative SGNH hydrolase-type esterase domain-containing protein [Medicago truncatula]	RHN81378.1	-5.45	1.16E-02	GO:0003674;GO:0003824;GO:0016787;GO:0016788	-
48 h	*MS.gene03596*	albumin-1 [Medicago truncatula]	XP_013459315.1	-5.5	1.16E-02	GO:0003674;GO:0008150;GO:0009405;GO:0044419;GO:0045735;GO:0051704	-
48 h	*MS.gene054182*	albumin-1 [Medicago truncatula]	XP_013452188.1	-5.55	1.44E-02	-	-
48 h	*MS.gene55847*	cytochrome P450 monooxygenase CYP716A12 [Medicago sativa]	AIT93302.1	-5.55	1.16E-02	GO:0003674;GO:0003824;GO:0005488;GO:0005506;GO:0008150;GO:0008152;GO:0016491;GO:0016705;GO:0020037;GO:0043167;GO:0043169;GO:0046872;GO:0046906;GO:0046914;GO:0048037;GO:0055114;GO:0097159;GO:1901363	K20667
48 h	*MS.gene021959*	hypothetical protein TSUD_170100 [Trifolium subterraneum]	GAU41899.1	-5.6	1.16E-02	-	-
48 h	*MS.gene84262*	putative ferric-chelate reductase (NADH) [Medicago truncatula]	RHN39861.1	-5.65	5.50E-03	GO:0003674;GO:0003824;GO:0008150;GO:0008152;GO:0016491;GO:0055114	K00521
48 h	*MS.gene47841*	beta-glucosidase 24 [Medicago truncatula]	XP_003604643.1	-6.01	1.16E-02	GO:0003674;GO:0003824;GO:0004553;GO:0005975;GO:0008150;GO:0008152;GO:0016787;GO:0016798;GO:0044238;GO:0071704	K01188
48 h	*MS.gene50073*	putative organ specific protein [Medicago truncatula]	RHN61275.1	-6.03	1.08E-03	-	-
48 h	*MS.gene033117*	GDSL esterase/lipase At1g74460 [Medicago truncatula]	XP_013457904.1	-6.1	1.70E-02	GO:0003674;GO:0003824;GO:0016787;GO:0016788	-
48 h	*MS.gene71626*	LOB domain-containing protein 38 [Medicago truncatula]	XP_003592478.1	-6.18	1.38E-02	-	-
48 h	*MS.gene50066*	hypothetical protein L195_g040127, partial [Trifolium pratense]	PNX84074.1	-6.21	1.70E-02	-	-
48 h	*MS.gene068715*	Fe(III) reduction oxidase [Medicago truncatula]	AQQ72609.1	-6.68	2.70E-02	GO:0003674;GO:0003824;GO:0008150;GO:0008152;GO:0016491;GO:0055114	-
48 h	*MS.gene06062*	hypothetical protein MTR_8g099365	KEH21207.1	-8.5	3.97E-02	-	-

		[Medicago truncatula]					
72 h	*MS.gene84367*	21 kDa protein [Medicago truncatula]	XP_003598238.1	6.75	9.13E-20	GO:0003674;GO:0004857;GO:0030234;GO:0098772	-
72 h	*MS.gene044034*	EF-hand pair protein [Medicago truncatula]	AES59455.1	6.53	1.08E-07	GO:0003674;GO:0005488;GO:0005509;GO:0043167;GO:0043169;GO:0046872	-
72 h	*MS.gene27308*	sugar transporter ERD6-like 6 [Medicago truncatula]	XP_013466113.1	6.04	1.97E-06	GO:0003674;GO:0005215;GO:0005575;GO:0006810;GO:0008150;GO:0016020;GO:0016021;GO:0022857;GO:0031224;GO:0051179;GO:0051234;GO:0055085;GO:0110165	K08145
72 h	*MS.gene91543*	Putative The fantastic four family protein[Medicago tribuloides]	XP_003591053.3	5.62	1.25E-04	-	-
72 h	*MS.gene06285*	ethylene-responsive transcription factor ERF114 [Medicago truncatula]	XP_003602747.1	5.58	4.47E-08	GO:0003674;GO:0003676;GO:0003677;GO:0003700;GO:0005488;GO:0006355;GO:0008150;GO:0009889;GO:0010468;GO:0010556;GO:0019219;GO:0019222;GO:0031323;GO:0031326;GO:0050789;GO:0050794;GO:0051171;GO:0051252;GO:0060255;GO:0065007;GO:0080090;GO:0097159;GO:0140110;GO:1901363;GO:1903506;GO:2000112;GO:2001141	-
72 h	*MS.gene074579*	albumin-1 [Medicago truncatula]	XP_003627386.2	5.5	1.37E-04	GO:0003674;GO:0008150;GO:0009405;GO:0044419;GO:0045735;GO:0051704	-
72 h	*MS.gene025233*	protein LURP-one-related 6 [Medicago truncatula]	XP_013441671.1	5.46	9.31E-08	-	-
72 h	*MS.gene059383*	probable RNA-binding protein 18 isoform X1 [Medicago truncatula]	XP_013452197.1	5.42	2.62E-04	GO:0003674;GO:0003676;GO:0005488;GO:0097159;GO:1901363	-
72 h	*MS.gene40367*	universal stress family protein [Medicago truncatula]	AES73813.2	5.37	2.94E-07	-	-
72 h	*MS.gene039268*	cytochrome P450 72A68-like [Medicago truncatula]	XP_013464434.2	5.34	3.92E-04	GO:0003674;GO:0003824;GO:0005488;GO:0005506;GO:0008150;GO:0008152;GO:0016491;GO:0016705;GO:0020037;GO:0043167;GO:0043169;GO:0046872;GO:0046906;GO:0046914;GO:0048037;GO:0055114;GO:0097159;GO:1901363	-
72 h	*MS.gene030228*	alpha-aminoadipic semialdehyde synthase isoform X2 [Medicago truncatula]	XP_003609930.2	5.3	3.23E-07	GO:0003674;GO:0003824;GO:0008150;GO:0008152;GO:0016491;GO:0055114	-
72 h	*MS.gene031167*	putative 7-deoxyloganetin glucosyltransferase [Medicago truncatula]	RHN50153.1	5.19	9.17E-04	GO:0003674;GO:0003824;GO:0016740;GO:0016757;GO:0016758	-
72 h	*MS.gene39967*	late embryogenesis abundant protein D-34 [Medicago truncatula]	XP_003590628.1	5.16	1.08E-03	-	-
72 h	*MS.gene024691*	UDP-glucose 4-epimerase GEPI48 isoform X1 [Medicago truncatula]	XP_003610979.1	5.13	2.10E-06	-	-
72 h	*MS.gene50167*	elicitor-responsive	XP_003616408.2	5.08	5.23E-04	-	-

		protein 1 [Medicago truncatula]					
72 h	*MS.gene30182*	uncharacterized protein LOC112422001 [Medicago truncatula]	XP_024640464.1	4.8	9.14E-05	GO:0003674;GO:0005488;GO:0008270;GO:0043167;GO:0043169;GO:0046872;GO:0046914	-
72 h	*MS.gene32039*	late embryogenesis abundant protein D-34 [Medicago truncatula]	XP_003590628.1	4.79	2.43E-21	-	-
72 h	*MS.gene002997*	STIG1-like protein [Medicago truncatula]	KEH36912.1	4.78	1.24E-15	-	-
72 h	*MS.gene29650*	probable 2-oxoglutarate-dependent dioxygenase At5g05600 [Medicago truncatula]	XP_003616646.1	4.67	3.63E-05	GO:0003674;GO:0003824;GO:0008150;GO:0008152;GO:0016491;GO:0055114	-
72 h	*MS.gene063320*	programmed cell death protein 4 [Medicago truncatula]	XP_003620790.1	-5.49	6.26E-10	-	-
72 h	*MS.gene058107*	ERAD-associated E3 ubiquitin-protein ligase HRD1B [Medicago truncatula]	XP_003610850.1	-5.49	6.66E-10	-	-
72 h	*MS.gene21760*	ERAD-associated E3 ubiquitin-protein ligase HRD1B [Medicago truncatula]	XP_003610850.1	-5.57	3.61E-07	-	K10601
72 h	*MS.gene40869*	hypothetical protein MTR_5g045310 [Medicago truncatula]	AES97098.2	-5.6	1.12E-10	-	K18635
72 h	*MS.gene016656*	hypothetical protein MtrunA17_Chr8g0352441 [Medicago truncatula]	RHN40223.1	-5.88	1.28E-12	-	-
72 h	*MS.gene020124*	disease resistance protein (TIR-NBS-LRR class), putative [Medicago truncatula]	AET01380.2	-5.92	9.35E-10	-	-
72 h	*MS.gene04888*	uncharacterized protein LOC112420870 [Medicago truncatula]	XP_024636395.1	-6.28	7.85E-12	GO:0000723;GO:0003674;GO:0003678;GO:0003824;GO:0004386;GO:0006139;GO:0006259;GO:0006281;GO:0006725;GO:0006807;GO:0006950;GO:0006974;GO:0006996;GO:0008094;GO:0008150;GO:0008152;GO:0009987;GO:0016043;GO:0016462;GO:0016787;GO:0016817;GO:0016818;GO:0016887;GO:0017111;GO:0032200;GO:0033554;GO:0034641;GO:0042592;GO:0042623;GO:0043170;GO:0044237;GO:0044238;GO:0044260;GO:0046483;GO:0050896;GO:0051276;GO:0051716;GO:0060249;GO:0065007;GO:0065008;GO:0071704;GO:0071840;GO:0090304;GO:0140097;GO:1901360	-

72 h	*MS.gene44226*	proteasome subunit alpha type-7 [Medicago truncatula]	XP_003629279.1	-6.53	1.13E-06	GO:0003674;GO:0003824;GO:0004175;GO:0004298;GO:0005575;GO:0005839;GO:0006508;GO:0006511;GO:0006807;GO:0008150;GO:0008152;GO:0008233;GO:0009056;GO:0009057;GO:0009987;GO:0016787;GO:0019538;GO:0019773;GO:0019941;GO:0032991;GO:0043170;GO:0043632;GO:0044237;GO:0044238;GO:0044248;GO:0044260;GO:0044265;GO:0051603;GO:0070003;GO:0070011;GO:0071704;GO:0140096;GO:1901564;GO:1901575	K02731
72 h	*MS.gene40444*	probable cyclic nucleotide-gated ion channel 20, chloroplastic [Medicago truncatula]	XP_013452813.1	-6.64	2.59E-08	GO:0003674;GO:0005215;GO:0005216;GO:0005575;GO:0006810;GO:0006811;GO:0008150;GO:0015075;GO:0015267;GO:0015318;GO:0016020;GO:0022803;GO:0022857;GO:0051179;GO:0051234;GO:0055085;GO:0110165	K05391
72 h	*MS.gene25760*	heat shock 70 kDa protein 17 [Medicago truncatula]	XP_013461130.1	-8.26	3.98E-06	-	K09486
72 h	*MS.gene051009*	elongation factor 1-alpha [Medicago truncatula]	AET01475.1	-8.98	1.61E-05	GO:0000166;GO:0001882;GO:0001883;GO:0003674;GO:0003824;GO:0003924;GO:0005488;GO:0005525;GO:0016462;GO:0016787;GO:0016817;GO:0016818;GO:0017076;GO:0017111;GO:0019001;GO:0032549;GO:0032550;GO:0032553;GO:0032555;GO:0032561;GO:0035639;GO:0036094;GO:0043167;GO:0043168;GO:0097159;GO:0097367;GO:1901265;GO:1901363	-
7 d	*MS.gene024774*	Chalcone synthase 6-4	P51079.1	11.49	4.41E-108	GO:0003674;GO:0003824;GO:0008150;GO:0008152;GO:0009058;GO:0016740;GO:0016746	-
7 d	*MS.gene07266*	hypothetical protein MTR_8g069135 [Medicago truncatula]	KEH20146.1	11.44	2.00E-16	-	-
7 d	*MS.gene26124*	glutamine synthetase nodule isozyme [Medicago truncatula]	XP_003616263.1	11.35	1.24E-20	GO:0003674;GO:0003824;GO:0004356;GO:0006082;GO:0006520;GO:0006541;GO:0006542;GO:0006807;GO:0008150;GO:0008152;GO:0008652;GO:0009058;GO:0009064;GO:0009084;GO:0009987;GO:0016053;GO:0016211;GO:0016874;GO:0016879;GO:0016880;GO:0019752;GO:0043436;GO:0044237;GO:0044238;GO:0044249;GO:0044281;GO:0044283;GO:0046394;GO:0071704;GO:1901564;GO:1901566;GO:1901576;GO:1901605;GO:1901607	K01915
7 d	*MS.gene065110*	cytochrome P450 CYP73A100 [Medicago truncatula]	XP_013470209.1	11.3	1.16E-57	GO:0003674;GO:0003824;GO:0005488;GO:0005506;GO:0008150;GO:0008152;GO:0016491;GO:0016705;GO:0020037;GO:0043167;GO:0043169;GO:0046872;GO:0046906;GO:0046914;GO:0048037;GO:0055114;GO:0097159;GO:1901363	-
7 d	*MS.gene034484*	elongation factor g [Trifolium pratense]	PNX95239.1	10.86	1.11E-156	-	-
7 d	*MS.gene37743*	kunitz trypsin inhibitor 5 [Medicago truncatula]	XP_003620188.1	10.75	1.62E-106	GO:0003674;GO:0004857;GO:0004866;GO:0030234;GO:0030414;GO:0061134;GO:0061135;GO:0098772	-
7 d	*MS.gene76234*	ferredoxin, root R-B1 isoform X1 [Medicago truncatula]	XP_013470266.1	10.54	1.23E-92	GO:0003674;GO:0003824;GO:0005488;GO:0006091;GO:0008150;GO:0008152;GO:0009055;GO:0009987;GO:0016491;GO:0022900;GO:0044237;GO:0048037;GO:0051536;GO:0051537;GO:0051540;GO:0055114	K02639
7 d	*MS.gene044942*	putative EF-hand domain pair protein [Medicago truncatula]	RHN44803.1	10.43	1.91E-88	GO:0003674;GO:0005488;GO:0005509;GO:0043167;GO:0043169;GO:0046872	-
7 d	*MS.gene005443*	putative XS domain-containing protein [Medicago truncatula]	RHN70044.1	10.35	5.67E-90	-	-
7 d	*MS.gene61263*	potassium	XP_013457577.1	10.34	8.11E-45	GO:0003674;GO:0005215;GO:0005575;GO:0006810;GO	K03549

		transporter 5 [Medicago truncatula]				:0006811;GO:0006812;GO:0006813;GO:0008150;GO:0008324;GO:0015075;GO:0015077;GO:0015079;GO:0015318;GO:0015672;GO:0016020;GO:0022857;GO:0022890;GO:0030001;GO:0034220;GO:0046873;GO:0051179;GO:0051234;GO:0055085;GO:0071805;GO:0098655;GO:0098660;GO:0098662;GO:0110165	
7 d	*MS.gene73420*	protein MAIN-LIKE 1-like [Medicago truncatula]	XP_024629818.1	10.33	3.51E-89	-	-
7 d	*MS.gene73019*	putative disease resistance protein At3g14460 [Medicago truncatula]	XP_003599073.1	10.3	4.19E-88	GO:0000166;GO:0003674;GO:0005488;GO:0017076;GO:0030554;GO:0032553;GO:0032555;GO:0032559;GO:0036094;GO:0043167;GO:0043168;GO:0043531;GO:0097159;GO:0097367;GO:1901265;GO:1901363	-
7 d	*MS.gene88029*	hypothetical protein TSUD_181560 [Trifolium subterraneum]	GAU49478.1	10.26	2.87E-86	-	-
7 d	*MS.gene98017*	F-box/FBD/LRR-repeat protein At1g13570 [Medicago truncatula]	XP_013463756.1	10.08	6.29E-121	GO:0003674;GO:0005488;GO:0005515	-
7 d	*MS.gene76574*	hypothetical protein MTR_2g100440 [Medicago truncatula]	AES67888.1	10.05	7.11E-48	-	-
7 d	*MS.gene028296*	WD-40 repeat-containing protein MSI1 [Medicago truncatula]	XP_003608509.1	10.04	2.62E-73	GO:0003674;GO:0005488;GO:0005515	-
7 d	*MS.gene86349*	uncharacterized protein LOC11433574 [Medicago truncatula]	XP_003597843.1	-10.13	3.36E-32	-	-
7 d	*MS.gene60716*	40S ribosomal protein S18 isoform X1 [Medicago truncatula]	XP_003592049.1	-10.14	2.31E-30	GO:0003674;GO:0003676;GO:0003723;GO:0003735;GO:0005198;GO:0005488;GO:0005575;GO:0005840;GO:0006412;GO:0006518;GO:0006807;GO:0008150;GO:0008152;GO:0009058;GO:0009059;GO:0009987;GO:0019538;GO:0034641;GO:0034645;GO:0043043;GO:0043170;GO:0043226;GO:0043228;GO:0043229;GO:0043232;GO:0043603;GO:0043604;GO:0044237;GO:0044238;GO:0044249;GO:0044260;GO:0044267;GO:0044271;GO:0071704;GO:0097159;GO:0110165;GO:1901363;GO:1901564;GO:1901566;GO:1901576	K02964
7 d	*MS.gene049580*	eukaryotic translation initiation factor 2 subunit beta [Medicago truncatula]	XP_013460480.1	-10.26	9.95E-101	GO:0003674;GO:0003676;GO:0003723;GO:0003743;GO:0005488;GO:0006413;GO:0008135;GO:0008150;GO:0008152;GO:0009987;GO:0044237;GO:0045182;GO:0090079;GO:0097159;GO:1901363	-
7 d	*MS.gene69251*	26S proteasome non-ATPase regulatory subunit 7 homolog A [Medicago truncatula]	XP_003600861.1	-10.3	2.45E-127	GO:0003674;GO:0005488;GO:0005515;GO:0005575;GO:0005838;GO:0032991	K03038
7 d	*MS.gene67404*	putative Extensin domain-containing protein [Medicago truncatula]	RHN50986.1	-10.55	3.40E-158	GO:0003674;GO:0005198;GO:0005199;GO:0005575;GO:0005576;GO:0008150;GO:0009664;GO:0009987;GO:0016043;GO:0045229;GO:0071554;GO:0071555;GO:0071669;GO:0071840;GO:0110165	-

7 d	*MS.gene69075*	Enod93 protein [Medicago sativa subsp. x varia]	CAB65283.1	-10.56	2.57E-76	-	-
7 d	*MS.gene027323*	40S ribosomal protein S8 [Medicago truncatula]	XP_013448528.1	-10.63	9.87E-128	GO:0003674;GO:0003735;GO:0005198;GO:0005575;GO:0005840;GO:0006412;GO:0006518;GO:0006807;GO:0008150;GO:0008152;GO:0009058;GO:0009059;GO:0009987;GO:0019538;GO:0034641;GO:0034645;GO:0043043;GO:0043170;GO:0043226;GO:0043228;GO:0043229;GO:0043232;GO:0043603;GO:0043604;GO:0044237;GO:0044238;GO:0044249;GO:0044260;GO:0044267;GO:0044271;GO:0071704;GO:0110165;GO:1901564;GO:1901566;GO:1901576	-
7 d	*MS.gene070073*	60S ribosomal protein L35a-1 [Medicago truncatula]	XP_013454243.1	-10.64	7.87E-112	GO:0003674;GO:0003735;GO:0005198;GO:0005575;GO:0005840;GO:0006412;GO:0006518;GO:0006807;GO:0008150;GO:0008152;GO:0009058;GO:0009059;GO:0009987;GO:0019538;GO:0034641;GO:0034645;GO:0043043;GO:0043170;GO:0043226;GO:0043228;GO:0043229;GO:0043232;GO:0043603;GO:0043604;GO:0044237;GO:0044238;GO:0044249;GO:0044260;GO:0044267;GO:0044271;GO:0071704;GO:0110165;GO:1901564;GO:1901566;GO:1901576	-
7 d	*MS.gene015447*	protein NRT1/ PTR FAMILY 6.3 [Medicago truncatula]	XP_003611286.1	-10.73	5.34E-97	GO:0003674;GO:0005215;GO:0005575;GO:0006810;GO:0008150;GO:0016020;GO:0022857;GO:0051179;GO:0051234;GO:0055085;GO:0110165	-
7 d	*MS.gene61470*	early nodulin-93 [Medicago truncatula]	XP_003609265.1	-10.8	2.66E-134	-	-
7 d	*MS.gene25760*	heat shock 70 kDa protein 17 [Medicago truncatula]	XP_013461130.1	-11.05	2.82E-109	-	K09486
7 d	*MS.gene068014*	uncharacterized protein C6C3.02c [Medicago truncatula]	XP_003608406.2	-11.19	5.85E-113	-	-
7 d	*MS.gene45689*	putative organ specific protein [Medicago truncatula]	RHN61275.1	-13.77	6.73E-104	-	-
7 d	*MS.gene99553*	MLP-like protein 28 [Medicago truncatula]	XP_013445246.1	-13.77	5.13E-70	GO:0006950;GO:0006952;GO:0008150;GO:0050896	-
Susceptible clonal lines							
Time	Gene ID	Putative function	NR ID[1]	CS vs TS		GO	KEGG
				log_2FC[2]	FDR[3]		
24 h	*MS.gene40202*	WD repeat-containing protein 11 isoform X1 [Medicago truncatula]	XP_003609372.2	-9.3	5.68E-37	-	-
24 h	*MS.gene06063*	uncharacterized protein LOC25502173 [Medicago truncatula]	XP_013447182.1	-9.31	3.49E-37	-	-
24 h	*MS.gene55599*	probable ADP-ribosylation factor GTPase-activating protein AGD14 [Medicago truncatula]	XP_013451499.1	-9.33	2.44E-25	GO:0003674;GO:0005096;GO:0008047;GO:0030234;GO:0030695;GO:0060589;GO:0098772	K15044
24 h	*MS.gene66984*	5-adenylylsulfate reductase-like protein [Medicago	AES75170.2	-9.36	1.80E-37	GO:0008150;GO:0009987;GO:0019725;GO:0042592;GO:0045454;GO:0050789;GO:0050794;GO:0065007;GO:0065008	-

		truncatula]					
24 h	*MS.gene057173*	AKIN gamma [Medicago truncatula]	AAO61674.1	-9.38	6.45E-11	-	-
24 h	*MS.gene38416*	senescence-associated carboxylesterase 101 isoform X1 [Medicago truncatula]	XP_013460462.1	-9.44	2.26E-53	GO:0006629;GO:0008150;GO:0008152;GO:0044238;GO:0071704	-
24 h	*MS.gene068014*	uncharacterized protein C6C3.02c [Medicago truncatula]	XP_003608406.2	-9.48	6.01E-62	-	-
24 h	*MS.gene36333*	transcription factor bHLH68 [Medicago truncatula]	XP_003597717.2	-9.49	7.03E-40	GO:0003674;GO:0005488;GO:0005515;GO:0046983	-
24 h	*MS.gene29714*	uncharacterized protein LOC11419949 [Medicago truncatula]	XP_024639058.1	-9.5	1.01E-31	-	-
24 h	*MS.gene57146*	cytochrome P450 82A3 [Medicago truncatula]	XP_003618363.1	-9.51	1.23E-21	GO:0003674;GO:0003824;GO:0005488;GO:0005506;GO:0008150;GO:0008152;GO:0016491;GO:0016705;GO:0020037;GO:0043167;GO:0043169;GO:0046872;GO:0046906;GO:0046914;GO:0048037;GO:0055114;GO:0097159;GO:1901363	K23136
24 h	*MS.gene59476*	pathogenesis-related thaumatin-like protein 3.5 [Medicago truncatula]	XP_003612186.1	-9.53	7.13E-29	-	-
24 h	*MS.gene071284*	peroxidase [Medicago sativa]	QCX35792.1	-9.61	4.67E-37	GO:0003674;GO:0003824;GO:0004601;GO:0005488;GO:0006950;GO:0006979;GO:0008150;GO:0008152;GO:0009056;GO:0009987;GO:0016209;GO:0016491;GO:0016684;GO:0016999;GO:0017001;GO:0017144;GO:0020037;GO:0042737;GO:0042743;GO:0042744;GO:0044237;GO:0044248;GO:0046906;GO:0048037;GO:0050896;GO:0051186;GO:0051187;GO:0055114;GO:0072593;GO:0097159;GO:1901363	-
24 h	*MS.gene49045*	serine/arginine-rich splicing factor RS40 isoform X1 [Medicago truncatula]	XP_003592315.2	-9.64	1.06E-34	GO:0003674;GO:0003676;GO:0005488;GO:0097159;GO:1901363	K12893
24 h	*MS.gene99614*	DUF674 family protein [Medicago truncatula]	KEH22914.1	-9.66	4.10E-38	-	-
24 h	*MS.gene71748*	hypothetical protein TSUD_50040 [Trifolium subterraneum]	GAU14792.1	-9.69	2.81E-19	GO:0000166;GO:0003674;GO:0003824;GO:0004672;GO:0005488;GO:0005524;GO:0006464;GO:0006468;GO:0006793;GO:0006796;GO:0006807;GO:0008144;GO:0008150;GO:0008152;GO:0009987;GO:0016301;GO:0016310;GO:0016740;GO:0016772;GO:0016773;GO:0017076;GO:0019538;GO:0030554;GO:0032553;GO:0032555;GO:0032559;GO:0035639;GO:0036094;GO:0036211;GO:0043167;GO:0043168;GO:0043170;GO:0043412;GO:0044237;GO:0044238;GO:0044260;GO:0044267;GO:0071704;GO:0097159;GO:0097367;GO:0140096;GO:1901265;GO:1901363;GO:1901564	-
24 h	*MS.gene030951*	guanosine nucleotide diphosphate dissociation inhibitor 1 [Medicago truncatula]	XP_003629947.1	-9.94	1.37E-25	GO:0003674;GO:0005092;GO:0005093;GO:0006810;GO:0007165;GO:0007264;GO:0008104;GO:0008150;GO:0009987;GO:0015031;GO:0015833;GO:0030234;GO:0030695;GO:0033036;GO:0035556;GO:0042886;GO:0045184;GO:0050789;GO:0050794;GO:0051179;GO:0051234;GO:0060589;GO:0065007;GO:0071702;GO:0071705;GO:0098772	-

24 h	*MS.gene055150*	probable glutathione S-transferase [Medicago truncatula]	XP_003623195.1	-9.98	1.43E-70	GO:0003674;GO:0005488;GO:0005515	-
24 h	*MS.gene24152*	snakin-2 [Medicago truncatula]	XP_003589486.1	-10.17	2.39E-05	-	-
24 h	*MS.gene70389*	uncharacterized protein LOC25493291 [Medicago truncatula]	XP_013457214.1	-10.2	1.38E-89	-	-
24 h	*MS.gene034179*	uncharacterized membrane protein At1g16860 isoform X1 [Medicago truncatula]	XP_013454562.1	-10.37	4.48E-53	-	-
24 h	*MS.gene47699*	mitochondrial fission 1 protein A [Medicago truncatula]	XP_003617291.1	-10.37	1.53E-28	GO:0000266;GO:0003674;GO:0005488;GO:0005515;GO:0006996;GO:0007005;GO:0008150;GO:0009987;GO:0016043;GO:0048285;GO:0071840	K17969
24 h	*MS.gene033016*	phenylalanine--tRNA ligase beta subunit, cytoplasmic [Medicago truncatula]	XP_003609165.2	-10.48	3.69E-42	GO:0000166;GO:0000287;GO:0003674;GO:0003676;GO:0003723;GO:0003824;GO:0004812;GO:0004826;GO:0005488;GO:0005524;GO:0005575;GO:0005737;GO:0006082;GO:0006139;GO:0006399;GO:0006418;GO:0006432;GO:0006520;GO:0006725;GO:0006807;GO:0008144;GO:0008150;GO:0008152;GO:0009987;GO:0016070;GO:0016874;GO:0016875;GO:0017076;GO:0019752;GO:0030554;GO:0032553;GO:0032555;GO:0032559;GO:0034641;GO:0034660;GO:0035639;GO:0036094;GO:0043038;GO:0043039;GO:0043167;GO:0043168;GO:0043169;GO:0043170;GO:0043436;GO:0044237;GO:0044238;GO:0044281;GO:0046483;GO:0046872;GO:0071704;GO:0090304;GO:0097159;GO:0097367;GO:0110165;GO:0140098;GO:0140101;GO:1901265;GO:1901360;GO:1901363;GO:1901564	-
24 h	*MS.gene036635*	uncharacterized protein LOC25493971 [Medicago truncatula]	XP_013458133.1	-10.54	4.42E-16	-	-
24 h	*MS.gene039293*	disease resistance protein RPP13-like isoform X1 [Vigna angularis]	XP_017431273.1	-10.56	3.04E-79	GO:0000166;GO:0003674;GO:0005488;GO:0017076;GO:0030554;GO:0032553;GO:0032555;GO:0032559;GO:0036094;GO:0043167;GO:0043168;GO:0043531;GO:0097159;GO:0097367;GO:1901265;GO:1901363	-
24 h	*MS.gene052116*	outer envelope pore protein 16, chloroplastic [Medicago truncatula]	XP_013465400.1	-10.59	1.63E-56	-	-
24 h	*MS.gene36325*	golgin candidate 5 [Medicago truncatula]	XP_003597734.2	-10.65	1.71E-46	-	K20286
24 h	*MS.gene035283*	CASP-like protein 1D2 [Medicago truncatula]	XP_003609582.1	-10.9	1.38E-39	-	-
24 h	*MS.gene038444*	protein RGF1 INDUCIBLE TRANSCRIPTION FACTOR 1 [Medicago truncatula]	XP_003628796.1	-11.11	3.88E-24	-	-
24 h	*MS.gene98726*	probable fructokinase-4	XP_003597523.1	-11.55	2.61E-82	GO:0003674;GO:0003824;GO:0016301;GO:0016740;GO:0016772;GO:0016773	-

24 h	*MS.gene61470*	[Medicago truncatula] early nodulin-93 [Medicago truncatula]	XP_003609265.1	-11.86	7.70E-30	-	-
48 h	*MS.gene72002*	Expansin-like B1[Arabidopsis thaliana]	AFK45939.1	9.44	2.59E-11	GO:0005575;GO:0005576;GO:0110165	-
48 h	*MS.gene069489*	miraculin [Medicago truncatula]	XP_003620184.1	8.57	9.44E-114	GO:0003674;GO:0004857;GO:0004866;GO:0030234;GO:0030414;GO:0061134;GO:0061135;GO:0098772	-
48 h	*MS.gene34631*	polygalacturonase inhibitor [Medicago truncatula]	XP_013445647.1	7.85	3.52E-48	GO:0003674;GO:0005488;GO:0005515	-
48 h	*MS.gene41339*	polygalacturonase inhibitor [Medicago truncatula]	XP_013467253.1	7.71	2.69E-19	GO:0003674;GO:0005488;GO:0005515	-
48 h	*MS.gene43364*	polygalacturonase inhibitor [Medicago truncatula]	XP_013467252.1	7.69	2.39E-31	-	-
48 h	*MS.gene068813*	polygalacturonase inhibitor [Medicago truncatula]	XP_013445647.1	7.28	4.01E-34	-	-
48 h	*MS.gene26988*	dihydroflavonol 4-reductase [Medicago sativa]	AEI59122.1	7.28	2.31E-27	GO:0003674;GO:0003824;GO:0005488;GO:0048037;GO:0050662	K13082
48 h	*MS.gene069444*	miraculin [Medicago truncatula]	XP_003620175.1	7.18	4.71E-53	GO:0003674;GO:0004857;GO:0004866;GO:0030234;GO:0030414;GO:0061134;GO:0061135;GO:0098772	-
48 h	*MS.gene64785*	thiosulfate sulfurtransferase 18 [Medicago truncatula]	XP_003612194.1	7.12	1.39E-10	-	-
48 h	*MS.gene069488*	miraculin [Medicago truncatula]	XP_003620185.1	6.86	8.43E-101	GO:0003674;GO:0004857;GO:0004866;GO:0030234;GO:0030414;GO:0061134;GO:0061135;GO:0098772	-
48 h	*MS.gene43357*	polygalacturonase inhibitor [Medicago truncatula]	XP_013467252.1	6.85	3.19E-28	-	-
48 h	*MS.gene037895*	polygalacturonase inhibitor [Medicago truncatula]	XP_013467252.1	6.77	9.64E-40	-	-
48 h	MS.gene068807	polygalacturonase inhibitor [Medicago truncatula]	XP_013445647.1	6.71	3.57E-40	-	-
48 h	MS.gene57814	miraculin [Medicago truncatula]	XP_003620185.1	6.64	3.74E-07	GO:0003674;GO:0004857;GO:0004866;GO:0030234;GO:0030414;GO:0061134;GO:0061135;GO:0098772	-
48 h	MS.gene037896	polygalacturonase inhibitor [Medicago truncatula]	XP_013467253.1	6.6	2.80E-21	GO:0003674;GO:0005488;GO:0005515	-
48 h	MS.gene05228	miraculin [Medicago truncatula]	XP_003620175.1	6.49	8.63E-21	GO:0003674;GO:0004857;GO:0004866;GO:0030234;GO:0030414;GO:0061134;GO:0061135;GO:0098772	-
48 h	MS.gene37741	miraculin [Medicago truncatula]	XP_003620185.1	6.45	8.02E-14	GO:0003674;GO:0004857;GO:0004866;GO:0030234;GO:0030414;GO:0061134;GO:0061135;GO:0098772	-
48 h	MS.gene41336	polygalacturonase	XP_013467252.1	6.35	4.67E-16	-	-

		inhibitor [Medicago truncatula]					
48 h	MS.gene011581	Expansin-like B1[Arabidopsis thaliana]	AFK45939.1	6.22	3.86E-91	GO:0005575;GO:0005576;GO:0110165	-
48 h	MS.gene72359	Expansin-like B1[Arabidopsis thaliana]	AFK45939.1	6.11	6.17E-04	GO:0005575;GO:0005576;GO:0110165	-
48 h	MS.gene68076	thiosulfate sulfurtransferase 18 [Medicago truncatula]	XP_003612194.1	6.06	1.25E-14	-	-
48 h	MS.gene006033	Isoliquiritigenin 2'-O-methyltransferase[Medicago sativa]	P93324.1	6.02	8.95E-20	GO:0003674;GO:0003824;GO:0005488;GO:0005515;GO:0008168;GO:0008171;GO:0016740;GO:0016741;GO:0046983	-
48 h	MS.gene006841	2-alkenal reductase (NADP(+)-dependent) [Medicago truncatula]	XP_003607791.1	5.81	1.39E-06	GO:0003674;GO:0003824;GO:0008150;GO:0008152;GO:0016491;GO:0055114	-
48 h	MS.gene71906	21 kDa protein [Medicago truncatula]	XP_003608771.2	5.79	1.32E-07	GO:0003674;GO:0004857;GO:0030234;GO:0098772	-
48 h	MS.gene57818	miraculin [Medicago truncatula]	XP_003620175.1	5.77	5.43E-11	GO:0003674;GO:0004857;GO:0004866;GO:0030234;GO:0030414;GO:0061134;GO:0061135;GO:0098772	-
48 h	MS.gene011579	Expansin-like B1[Arabidopsis thaliana]	AFK45939.1	5.67	2.03E-38	GO:0005575;GO:0005576;GO:0110165	-
48 h	MS.gene073143	protein SENSITIVE TO PROTON RHIZOTOXICITY 2-like [Medicago truncatula]	XP_024631543.1	-5.38	7.64E-07	GO:0003674;GO:0003676;GO:0005488;GO:0097159;GO:1901363	-
72 h	MS.gene77495	phytoene synthase 2, chloroplastic [Medicago truncatula]	XP_003601619.1	8.46	1.04E-43	GO:0003674;GO:0003824;GO:0004310;GO:0004311;GO:0004659;GO:0006066;GO:0006629;GO:0006694;GO:0006696;GO:0008150;GO:0008152;GO:0008202;GO:0008204;GO:0008610;GO:0009058;GO:0009987;GO:0016125;GO:0016126;GO:0016128;GO:0016129;GO:0016740;GO:0016765;GO:0044107;GO:0044108;GO:0044237;GO:0044238;GO:0044249;GO:0044255;GO:0044281;GO:0044283;GO:0046165;GO:0051996;GO:0071704;GO:0097384;GO:1901360;GO:1901362;GO:1901576;GO:1901615;GO:1901617;GO:1902652;GO:1902653	K02291
72 h	MS.gene062981	glutelin type-D 1 isoform X1 [Medicago truncatula]	XP_013456078.1	7.35	2.57E-23	GO:0003674;GO:0045735	-
72 h	MS.gene74031	polygalacturonase inhibitor 1-like [Medicago truncatula]	XP_024625848.1	7.31	7.58E-03	GO:0003674;GO:0005488;GO:0005515	-
72 h	MS.gene009912	putative glucan 1,3-beta-glucosidase [Medicago truncatula]	RHN56441.1	7.26	5.47E-19	GO:0003674;GO:0003824;GO:0004553;GO:0005975;GO:0008150;GO:0008152;GO:0016787;GO:0016798;GO:0044238;GO:0071704	-
72 h	MS.gene93158	F-box SKP2A-like protein [Medicago truncatula]	KEH40480.1	7.19	5.59E-36	GO:0003674;GO:0005488;GO:0005515	K03875
72 h	MS.gene47489	uncharacterized protein	XP_003608032.1	6.95	6.95E-10	GO:0003674;GO:0003824;GO:0009055;GO:0016491	-

72 h	MS.gene069489	LOC11441412 [Medicago truncatula] miraculin [Medicago truncatula]	XP_003620184.1	6.81	3.78E-42	GO:0003674;GO:0004857;GO:0004866;GO:0030234;GO:0030414;GO:0061134;GO:0061135;GO:0098772	-
72 h	MS.gene004947	alpha-galactosidase [Medicago truncatula]	XP_013468838.1	6.77	6.57E-13	GO:0003674;GO:0003824;GO:0004553;GO:0005975;GO:0008150;GO:0008152;GO:0016787;GO:0016798;GO:0044238;GO:0071704	-
72 h	MS.gene069172	ABC transporter G family member STR2 [Medicago truncatula]	XP_003612949.1	6.76	1.20E-13	GO:0000166;GO:0003674;GO:0003824;GO:0005488;GO:0005524;GO:0005575;GO:0008144;GO:0016020;GO:0016462;GO:0016787;GO:0016817;GO:0016818;GO:0016887;GO:0017076;GO:0017111;GO:0030554;GO:0032553;GO:0032555;GO:0032559;GO:0035639;GO:0036094;GO:0043167;GO:0043168;GO:0097159;GO:0097367;GO:0110165;GO:1901265;GO:1901363	-
72 h	MS.gene05113	60S ribosomal protein L10 [Medicago truncatula]	XP_003600557.1	6.74	2.16E-13	GO:0003674;GO:0003735;GO:0005198;GO:0005575;GO:0005840;GO:0006412;GO:0006518;GO:0006807;GO:0008150;GO:0008152;GO:0009058;GO:0009059;GO:0009987;GO:0019538;GO:0034641;GO:0034645;GO:0043043;GO:0043170;GO:0043226;GO:0043228;GO:0043229;GO:0043232;GO:0043603;GO:0043604;GO:0044237;GO:0044238;GO:0044249;GO:0044260;GO:0044267;GO:0044271;GO:0071704;GO:0110165;GO:1901564;GO:1901566;GO:1901576	K02866
72 h	MS.gene054616	Putative cupredoxin[Medicago truncatula]	XP_003602997.3	6.65	9.24E-13	GO:0003674;GO:0003824;GO:0009055;GO:0016491	-
72 h	MS.gene035612	hypothetical protein TSUD_201850 [Trifolium subterraneum]	GAU18298.1	6.61	5.08E-10	-	-
72 h	MS.gene54097	probable E3 ubiquitin-protein ligase RZFP34 isoform X2 [Medicago truncatula]	XP_024630999.1	6.48	1.35E-07	GO:0003674;GO:0005488;GO:0008270;GO:0043167;GO:0043169;GO:0046872;GO:0046914	K10144
72 h	MS.gene031529	thioredoxin-like protein CXXS1 [Medicago truncatula]	XP_003607703.1	6.28	2.87E-04	GO:0008150;GO:0009987;GO:0019725;GO:0042592;GO:0045454;GO:0050789;GO:0050794;GO:0065007;GO:0065008	-
72 h	MS.gene049015	transcription factor MYBS3 [Medicago truncatula]	XP_003630638.1	6.26	9.37E-10	GO:0003674;GO:0003676;GO:0003677;GO:0005488;GO:0097159;GO:1901363	-
72 h	MS.gene02350	stigma-specific STIG1-like protein 1 [Medicago truncatula]	XP_013462876.1	6.24	7.47E-04	-	-
72 h	MS.gene20167	transcription factor MYBS3 [Medicago truncatula]	XP_003630638.1	6.19	4.42E-23	GO:0003674;GO:0003676;GO:0003677;GO:0005488;GO:0097159;GO:1901363	-
72 h	MS.gene013138	F-box protein At5g50450 [Medicago truncatula]	XP_003602817.2	6.19	1.45E-08	GO:0003674;GO:0005488;GO:0005515	-
72 h	MS.gene05233	miraculin [Medicago truncatula]	XP_003620187.1	6.18	2.99E-09	GO:0003674;GO:0004857;GO:0004866;GO:0030234;GO:0030414;GO:0061134;GO:0061135;GO:0098772	-
72 h	MS.gene055509	translation initiation factor IF-2-like protein [Trifolium	PNY16350.1	6.14	5.26E-06	GO:0003674;GO:0005488;GO:0006810;GO:0006811;GO:0006812;GO:0008150;GO:0030001;GO:0043167;GO:0043169;GO:0046872;GO:0051179;GO:0051234	-

		pratense]					
72 h	MS.gene01384	serine carboxypeptidase-like 32 [Medicago truncatula]	XP_003593501.2	6.04	6.65E-38	GO:0003674;GO:0003824;GO:0004180;GO:0004185;GO:0006508;GO:0006807;GO:0008150;GO:0008152;GO:0008233;GO:0008236;GO:0008238;GO:0016787;GO:0016825;GO:0017171;GO:0019538;GO:0043170;GO:0044238;GO:0070008;GO:0070011;GO:0071704;GO:0140096;GO:1901564	K16297
72 h	MS.gene009912	putative glucan 1,3-beta-glucosidase [Medicago truncatula]	RHN56441.1	7.26	5.47E-19	GO:0003674;GO:0003824;GO:0004553;GO:0005975;GO:0008150;GO:0008152;GO:0016787;GO:0016798;GO:0044238;GO:0071704	-
72 h	MS.gene93158	F-box SKP2A-like protein [Medicago truncatula]	KEH40480.1	7.19	5.59E-36	GO:0003674;GO:0005488;GO:0005515	K03875
72 h	MS.gene47489	uncharacterized protein LOC11441412 [Medicago truncatula]	XP_003608032.1	6.95	6.95E-10	GO:0003674;GO:0003824;GO:0009055;GO:0016491	-
72 h	MS.gene069489	miraculin [Medicago truncatula]	XP_003620184.1	6.81	3.78E-42	GO:0003674;GO:0004857;GO:0004866;GO:0030234;GO:0030414;GO:0061134;GO:0061135;GO:0098772	-
72 h	MS.gene004947	alpha-galactosidase [Medicago truncatula]	XP_013468838.1	6.77	6.57E-13	GO:0003674;GO:0003824;GO:0004553;GO:0005975;GO:0008150;GO:0008152;GO:0016787;GO:0016798;GO:0044238;GO:0071704	-
72 h	MS.gene069172	ABC transporter G family member STR2 [Medicago truncatula]	XP_003612949.1	6.76	1.20E-13	GO:0000166;GO:0003674;GO:0003824;GO:0005488;GO:0005524;GO:0005575;GO:0008144;GO:0016020;GO:0016462;GO:0016787;GO:0016817;GO:0016818;GO:0016887;GO:0017076;GO:0017111;GO:0030554;GO:0032553;GO:0032555;GO:0032559;GO:0035639;GO:0036094;GO:0043167;GO:0043168;GO:0097159;GO:0097367;GO:0110165;GO:1901265;GO:1901363	-
72 h	MS.gene05113	60S ribosomal protein L10 [Medicago truncatula]	XP_003600557.1	6.74	2.16E-13	GO:0003674;GO:0003735;GO:0005198;GO:0005575;GO:0005840;GO:0006412;GO:0006518;GO:0006807;GO:0008150;GO:0008152;GO:0009058;GO:0009059;GO:0009987;GO:0019538;GO:0034641;GO:0034645;GO:0043043;GO:0043170;GO:0043226;GO:0043228;GO:0043229;GO:0043232;GO:0043603;GO:0043604;GO:0044237;GO:0044238;GO:0044249;GO:0044260;GO:0044267;GO:0044271;GO:0071704;GO:0110165;GO:1901564;GO:1901566;GO:1901576	K02866
72 h	MS.gene054616	Putative cupredoxin[Medicago truncatula]	XP_003602997.3	6.65	9.24E-13	GO:0003674;GO:0003824;GO:0009055;GO:0016491	-
72 h	MS.gene035612	hypothetical protein TSUD_201850 [Trifolium subterraneum]	GAU18298.1	6.61	5.08E-10	-	-
72 h	MS.gene54097	probable E3 ubiquitin-protein ligase RZFP34 isoform X2 [Medicago truncatula]	XP_024630999.1	6.48	1.35E-07	GO:0003674;GO:0005488;GO:0008270;GO:0043167;GO:0043169;GO:0046872;GO:0046914	K10144
72 h	MS.gene031529	thioredoxin-like protein CXXS1 [Medicago truncatula]	XP_003607703.1	6.28	2.87E-04	GO:0008150;GO:0009987;GO:0019725;GO:0042592;GO:0045454;GO:0050789;GO:0050794;GO:0065007;GO:0065008	-
72 h	MS.gene049015	transcription factor MYBS3 [Medicago truncatula]	XP_003630638.1	6.26	9.37E-10	GO:0003674;GO:0003676;GO:0003677;GO:0005488;GO:0097159;GO:1901363	-
72 h	MS.gene02350	stigma-specific STIG1-like protein 1	XP_013462876.1	6.24	7.47E-04	-	-

		[Medicago truncatula]					
72 h	MS.gene20167	transcription factor MYBS3 [Medicago truncatula]	XP_003630638.1	6.19	4.42E-23	GO:0003674;GO:0003676;GO:0003677;GO:0005488;GO:0097159;GO:1901363	-
72 h	MS.gene013138	F-box protein At5g50450 [Medicago truncatula]	XP_003602817.2	6.19	1.45E-08	GO:0003674;GO:0005488;GO:0005515	-
72 h	MS.gene05233	miraculin [Medicago truncatula]	XP_003620187.1	6.18	2.99E-09	GO:0003674;GO:0004857;GO:0004866;GO:0030234;GO:0030414;GO:0061134;GO:0061135;GO:0098772	-
72 h	MS.gene055509	translation initiation factor IF-2-like protein [Trifolium pratense]	PNY16350.1	6.14	5.26E-06	GO:0003674;GO:0005488;GO:0006810;GO:0006811;GO:0006812;GO:0008150;GO:0030001;GO:0043167;GO:0043169;GO:0046872;GO:0051179;GO:0051234	-
72 h	MS.gene01384	serine carboxypeptidase-like 32 [Medicago truncatula]	XP_003593501.2	6.04	6.65E-38	GO:0003674;GO:0003824;GO:0004180;GO:0004185;GO:0006508;GO:0006807;GO:0008150;GO:0008152;GO:0008233;GO:0008236;GO:0008238;GO:0016787;GO:0016825;GO:0017171;GO:0019538;GO:0043170;GO:0044238;GO:0070008;GO:0070011;GO:0071704;GO:0140096;GO:1901564	K16297
72 h	MS.gene28804	hypothetical protein L195_g045964 [Trifolium pratense]	PNX89842.1	6.03	1.84E-05	-	-
72 h	MS.gene059867	NAC2 transcription factor [Medicago sativa]	QDC33485.1	6.02	1.20E-88	GO:0003674;GO:0003676;GO:0003677;GO:0005488;GO:0006355;GO:0008150;GO:0009889;GO:0010468;GO:0010556;GO:0019219;GO:0019222;GO:0031323;GO:0031326;GO:0050789;GO:0050794;GO:0051171;GO:0051252;GO:0060255;GO:0065007;GO:0080090;GO:0097159;GO:1901363;GO:1903506;GO:2000112;GO:2001141	-
72 h	MS.gene045577	eukaryotic translation initiation factor eIF2A-like protein [Medicago truncatula]	AES90259.1	-6.07	2.30E-14	-	-
72 h	MS.gene57110	uncharacterized protein Mb2253c-like [Medicago truncatula]	XP_024641091.1	-6.25	1.06E-15	GO:0003674;GO:0003676;GO:0003824;GO:0004518;GO:0004519;GO:0004521;GO:0004523;GO:0004540;GO:0005488;GO:0016787;GO:0016788;GO:0016891;GO:0016893;GO:0097159;GO:0140098;GO:1901363	-
72 h	MS.gene031303	putative lyase [Medicago truncatula]	RHN75244.1	-6.48	3.64E-19	GO:0000287;GO:0003674;GO:0003824;GO:0005488;GO:0010333;GO:0016829;GO:0016835;GO:0016838;GO:0043167;GO:0043169;GO:0046872	-
72 h	MS.gene008043	60S ribosomal protein L10a-1 [Medicago truncatula]	XP_003600923.1	-6.68	1.63E-21	GO:0003674;GO:0003676;GO:0003723;GO:0003735;GO:0005198;GO:0005488;GO:0005575;GO:0006412;GO:0006518;GO:0006807;GO:0008150;GO:0008152;GO:0009058;GO:0009059;GO:0009987;GO:0015934;GO:0019538;GO:0032991;GO:0034641;GO:0034645;GO:0043043;GO:0043170;GO:0043603;GO:0043604;GO:0044237;GO:0044238;GO:0044249;GO:0044260;GO:0044267;GO:0044271;GO:0044391;GO:0071704;GO:0097159;GO:1901363;GO:1901564;GO:1901566;GO:1901576;GO:1990904	-
72 h	MS.gene30023	Leucine--tRNA ligase, cytoplasmic isoform D [Glycine soja]	RZB78133.1	-6.76	6.07E-39	GO:0002161;GO:0003674;GO:0003824;GO:0006082;GO:0006139;GO:0006399;GO:0006418;GO:0006520;GO:0006725;GO:0006807;GO:0008150;GO:0008152;GO:0009987;GO:0016070;GO:0016787;GO:0016788;GO:0019752;GO:0034641;GO:0034660;GO:0043038;GO:0043039;GO:0043170;GO:0043436;GO:0044237;GO:0044238;GO:0044281;GO:0046483;GO:0052689;GO:0071704;GO:0090304;GO:0140098;GO:0140101;GO:1901360;GO:1901564	K01869
72 h	MS.gene01087	albumin-2	XP_003625763.1	-7.12	1.26E-20	-	-

	7	[Medicago truncatula]					
72 h	MS.gene83692	RALF-like protein [Medicago truncatula]	KEH29493.1	-7.35	1.95E-55	-	-
72 h	MS.gene01384	serine carboxypeptidase-like 32 [Medicago truncatula]	XP_003593501.2	6.04	6.65E-38	GO:0003674;GO:0003824;GO:0004180;GO:0004185;GO:0006508;GO:0006807;GO:0008150;GO:0008152;GO:0008233;GO:0008236;GO:0008238;GO:0016787;GO:0016825;GO:0017171;GO:0019538;GO:0043170;GO:0044238;GO:0070008;GO:0070011;GO:0071704;GO:0140096;GO:1901564	K16297
72 h	MS.gene28804	hypothetical protein L195_g045964 [Trifolium pratense]	PNX89842.1	6.03	1.84E-05	-	-
72 h	MS.gene059867	NAC2 transcription factor [Medicago sativa]	QDC33485.1	6.02	1.20E-88	GO:0003674;GO:0003676;GO:0003677;GO:0005488;GO:0006355;GO:0008150;GO:0009889;GO:0010468;GO:0010556;GO:0019219;GO:0019222;GO:0031323;GO:0031326;GO:0050789;GO:0050794;GO:0051171;GO:0051252;GO:0060255;GO:0065007;GO:0080090;GO:0097159;GO:1901363;GO:1903506;GO:2000112;GO:2001141	-
72 h	MS.gene045577	eukaryotic translation initiation factor eIF2A-like protein [Medicago truncatula]	AES90259.1	-6.07	2.30E-14	-	-
72 h	MS.gene57110	uncharacterized protein Mb2253c-like [Medicago truncatula]	XP_024641091.1	-6.25	1.06E-15	GO:0003674;GO:0003676;GO:0003824;GO:0004518;GO:0004519;GO:0004521;GO:0004523;GO:0004540;GO:0005488;GO:0016787;GO:0016788;GO:0016891;GO:0016893;GO:0097159;GO:0140098;GO:1901363	-
72 h	MS.gene031303	putative lyase [Medicago truncatula]	RHN75244.1	-6.48	3.64E-19	GO:0000287;GO:0003674;GO:0003824;GO:0005488;GO:0010333;GO:0016829;GO:0016835;GO:0016838;GO:0043167;GO:0043169;GO:0046872	-
72 h	MS.gene008043	60S ribosomal protein L10a-1 [Medicago truncatula]	XP_003600923.1	-6.68	1.63E-21	GO:0003674;GO:0003676;GO:0003723;GO:0003735;GO:0005198;GO:0005488;GO:0005575;GO:0006412;GO:0006518;GO:0006807;GO:0008150;GO:0008152;GO:0009058;GO:0009059;GO:0009987;GO:0015934;GO:0019538;GO:0032991;GO:0034641;GO:0034645;GO:0043043;GO:0043170;GO:0043603;GO:0043604;GO:0044237;GO:0044238;GO:0044249;GO:0044260;GO:0044267;GO:0044271;GO:0044391;GO:0071704;GO:0097159;GO:1901363;GO:1901564;GO:1901566;GO:1901576;GO:1990904	-
72 h	MS.gene30023	Leucine--tRNA ligase, cytoplasmic isoform D [Glycine soja]	RZB78133.1	-6.76	6.07E-39	GO:0002161;GO:0003674;GO:0003824;GO:0006082;GO:0006139;GO:0006399;GO:0006418;GO:0006520;GO:0006725;GO:0006807;GO:0008150;GO:0008152;GO:0009987;GO:0016070;GO:0016787;GO:0016788;GO:0019752;GO:0034641;GO:0034660;GO:0043038;GO:0043039;GO:0043170;GO:0043436;GO:0044237;GO:0044238;GO:0044281;GO:0046483;GO:0052689;GO:0071704;GO:0090304;GO:0140098;GO:0140101;GO:1901360;GO:1901564	K01869
72 h	MS.gene010877	albumin-2 [Medicago truncatula]	XP_003625763.1	-7.12	1.26E-20	-	-
72 h	MS.gene83692	RALF-like protein [Medicago truncatula]	KEH29493.1	-7.35	1.95E-55	-	-
72 h	MS.gene01384	serine carboxypeptidase-like 32 [Medicago truncatula]	XP_003593501.2	6.04	6.65E-38	GO:0003674;GO:0003824;GO:0004180;GO:0004185;GO:0006508;GO:0006807;GO:0008150;GO:0008152;GO:0008233;GO:0008236;GO:0008238;GO:0016787;GO:0016825;GO:0017171;GO:0019538;GO:0043170;GO:0044238;GO:0070008;GO:0070011;GO:0071704;GO:0140096;GO:1901564	K16297
7 d	MS.gene01474	guanine	XP_019417257.1	9.93	3.58E-46	GO:0007165;GO:0007186;GO:0008150;GO:0009987;GO	-

	7	nucleotide-binding protein subunit gamma 2-like isoform X1 [Lupinus angustifolius]				:0050789;GO:0050794;GO:0065007	
7 d	MS.gene24152	snakin-2 [Medicago truncatula]	XP_003589486.1	-8.42	8.96E-10	-	-
7 d	MS.gene51387	5' exonuclease Apollo [Medicago truncatula]	XP_013467578.1	-8.44	4.50E-10	-	K15341
7 d	MS.gene80107	uncharacterized protein LOC11435965 [Medicago truncatula]	XP_003601452.1	-8.46	9.51E-08	-	-
7 d	MS.gene67663	protein OS-9 homolog [Medicago truncatula]	XP_013463861.1	-8.46	7.27E-24	-	K10088
7 d	MS.gene38416	senescence-associated carboxylesterase 101 isoform X1 [Medicago truncatula]	XP_013460462.1	-8.47	3.87E-13	GO:0006629;GO:0008150;GO:0008152;GO:0044238;GO:0071704	-
7 d	MS.gene002152	actin-101 [Glycine max]	NP_001345025.1	-8.49	1.42E-57	-	-
7 d	MS.gene06598	S-adenosyl-L-methionine: caffeic acid 3-0-methyltransferase [Medicago sativa]	ACY06328.1	-8.49	1.60E-17	GO:0003674;GO:0003824;GO:0005488;GO:0005515;GO:0008168;GO:0008171;GO:0016740;GO:0016741;GO:0046983	K13066
7 d	MS.gene47699	mitochondrial fission 1 protein A [Medicago truncatula]	XP_003617291.1	-8.5	5.90E-47	GO:0000266;GO:0003674;GO:0005488;GO:0005515;GO:0006996;GO:0007005;GO:0008150;GO:0009987;GO:0016043;GO:0048285;GO:0071840	K17969
7 d	MS.gene85245	threonine synthase, chloroplastic [Medicago truncatula]	XP_003597553.1	-8.65	1.75E-44	GO:0003674;GO:0005488;GO:0006082;GO:0006520;GO:0006807;GO:0008144;GO:0008150;GO:0008152;GO:0009987;GO:0019752;GO:0019842;GO:0030170;GO:0036094;GO:0043167;GO:0043168;GO:0043436;GO:0044237;GO:0044238;GO:0044281;GO:0048037;GO:0050662;GO:0070279;GO:0071704;GO:0097159;GO:1901363;GO:1901564	K01733
7 d	MS.gene044049	serine/threonine-protein kinase tricornered isoform X1 [Medicago truncatula]	XP_003589178.1	-8.69	1.38E-27	GO:0000166;GO:0003674;GO:0003824;GO:0004672;GO:0004674;GO:0005488;GO:0005524;GO:0006464;GO:0006468;GO:0006793;GO:0006796;GO:0006807;GO:0008144;GO:0008150;GO:0008152;GO:0009987;GO:0016301;GO:0016310;GO:0016740;GO:0016772;GO:0016773;GO:0017076;GO:0019538;GO:0030554;GO:0032553;GO:0032555;GO:0032559;GO:0035639;GO:0036094;GO:0036211;GO:0043167;GO:0043168;GO:0043170;GO:0043412;GO:0044237;GO:0044238;GO:0044260;GO:0044267;GO:0071704;GO:0097159;GO:0097367;GO:0140096;GO:1901265;GO:1901363;GO:1901564	-
7 d	MS.gene051413	type-1 glutamine synthetase 1 [Medicago truncatula]	XP_003599597.1	-8.72	6.95E-12	GO:0003674;GO:0003824;GO:0004356;GO:0006082;GO:0006520;GO:0006541;GO:0006542;GO:0006807;GO:0008150;GO:0008152;GO:0008652;GO:0009058;GO:0009064;GO:0009084;GO:0009987;GO:0016053;GO:0016211;GO:0016874;GO:0016879;GO:0016880;GO:0019752;GO:0043436;GO:0044237;GO:0044238;GO:0044249;GO:0044281;GO:0044283;GO:0046394;GO:0071704;GO:1901564;GO:1901566;GO:1901576;GO:1901605;GO:1901607	-
7 d	MS.gene070361	threonine--tRNA ligase,	XP_003613276.1	-8.8	1.20E-16	GO:0000166;GO:0003674;GO:0003824;GO:0004812;GO:0005488;GO:0005515;GO:0005524;GO:0006082;GO:00	-

		mitochondrial 1 [Medicago truncatula]				06139;GO:0006399;GO:0006520;GO:0006725;GO:0006807;GO:0008144;GO:0008150;GO:0008152;GO:0009987;GO:0016070;GO:0016874;GO:0016875;GO:0017076;GO:0019752;GO:0030554;GO:0032553;GO:0032555;GO:0032559;GO:0034641;GO:0034660;GO:0035639;GO:0036094;GO:0043038;GO:0043039;GO:0043167;GO:0043168;GO:0043170;GO:0043436;GO:0044237;GO:0044238;GO:0044281;GO:0046483;GO:0071704;GO:0090304;GO:0097159;GO:0097367;GO:0140098;GO:0140101;GO:1901265;GO:1901360;GO:1901363;GO:1901564	
7 d	MS.gene031451	probable bifunctional methylthioribulose-1-phosphate dehydratase/enolase-phosphatase E1 [Medicago truncatula]	XP_013464689.1	-8.8	1.04E-13	GO:0000096;GO:0000097;GO:0000287;GO:0003674;GO:0003824;GO:0005488;GO:0005575;GO:0005737;GO:0006082;GO:0006520;GO:0006555;GO:0006790;GO:0006807;GO:0008150;GO:0008152;GO:0008652;GO:0009058;GO:0009066;GO:0009067;GO:0009086;GO:0009987;GO:0016053;GO:0016787;GO:0016788;GO:0016791;GO:0017144;GO:0019509;GO:0019752;GO:0042578;GO:0043094;GO:0043102;GO:0043167;GO:0043169;GO:0043436;GO:0043874;GO:0044237;GO:0044238;GO:0044249;GO:0044272;GO:0044281;GO:0044283;GO:0046394;GO:0046872;GO:0071265;GO:0071267;GO:0071704;GO:0110165;GO:1901564;GO:1901566;GO:1901576;GO:1901605;GO:1901607	-
7 d	MS.gene035283	CASP-like protein 1D2 [Medicago truncatula]	XP_003609582.1	-8.8	1.02E-06	-	-
7 d	MS.gene98729	electron transfer flavoprotein-ubiquinone oxidoreductase, mitochondrial isoform X2 [Medicago truncatula]	XP_024632579.1	-8.95	1.34E-07	-	-
7 d	MS.gene36615	probable LRR receptor-like serine/threonine-protein kinase At1g05700 [Medicago truncatula]	XP_003627069.2	-8.99	1.19E-12	GO:0000166;GO:0003674;GO:0003824;GO:0004672;GO:0005488;GO:0005515;GO:0005524;GO:0006464;GO:0006468;GO:0006793;GO:0006796;GO:0006807;GO:0008144;GO:0008150;GO:0008152;GO:0009987;GO:0016301;GO:0016310;GO:0016740;GO:0016772;GO:0016773;GO:0017076;GO:0019538;GO:0030554;GO:0032553;GO:0032555;GO:0032559;GO:0035639;GO:0036094;GO:0036211;GO:0043167;GO:0043168;GO:0043170;GO:0043412;GO:0044237;GO:0044238;GO:0044260;GO:0044267;GO:0071704;GO:0097159;GO:0097367;GO:0140096;GO:1901265;GO:1901363;GO:1901564	-
7 d	MS.gene30945	probable histone H2A.2 [Medicago truncatula]	XP_003597343.1	-9.06	1.28E-24	GO:0000786;GO:0003674;GO:0003676;GO:0003677;GO:0005488;GO:0005515;GO:0005575;GO:0005634;GO:0032991;GO:0032993;GO:0043226;GO:0043227;GO:0043229;GO:0043231;GO:0044815;GO:0046982;GO:0046983;GO:0097159;GO:0110165;GO:1901363	K11251
7 d	MS.gene03600	albumin-1 [Medicago truncatula]	XP_013459315.1	-9.09	9.96E-10	GO:0003674;GO:0008150;GO:0009405;GO:0044419;GO:0045735;GO:0051704	-
7 d	MS.gene054182	albumin-1 [Medicago truncatula]	XP_013452188.1	-9.19	2.96E-37	-	-
7 d	MS.gene051009	elongation factor 1-alpha [Medicago truncatula]	AET01475.1	-9.32	1.46E-29	GO:0000166;GO:0001882;GO:0001883;GO:0003674;GO:0003824;GO:0003924;GO:0005488;GO:0005525;GO:0016462;GO:0016787;GO:0016817;GO:0016818;GO:0017076;GO:0017111;GO:0019001;GO:0032549;GO:0032550;GO:0032553;GO:0032555;GO:0032561;GO:0035639;GO:0036094;GO:0043167;GO:0043168;GO:0097159;GO:0097367;GO:1901265;GO:1901363	-
7 d	MS.gene98726	probable fructokinase-4 [Medicago truncatula]	XP_003597523.1	-9.33	1.67E-32	GO:0003674;GO:0003824;GO:0016301;GO:0016740;GO:0016772;GO:0016773	-

7 d	MS.gene70389	uncharacterized protein LOC25493291 [Medicago truncatula]	XP_013457214.1	-9.56	3.45E-21	-	-
7 d	MS.gene36325	golgin candidate 5 [Medicago truncatula]	XP_003597734.2	-9.61	1.98E-19	-	K20286
7 d	MS.gene038444	protein RGF1 INDUCIBLE TRANSCRIPTION FACTOR 1 [Medicago truncatula]	XP_003628796.1	-9.69	6.19E-06	-	-
7 d	MS.gene051702	beta-amyrin 24-hydroxylase [Medicago truncatula]	XP_013448592.1	-9.72	3.03E-12	GO:0003674;GO:0003824;GO:0005488;GO:0005506;GO:0008150;GO:0008152;GO:0016491;GO:0016705;GO:0020037;GO:0043167;GO:0043169;GO:0046872;GO:0046906;GO:0046914;GO:0048037;GO:0055114;GO:0097159;GO:1901363	-
7 d	MS.gene052705	linoleate 9S-lipoxygenase [Medicago truncatula]	XP_003627189.1	-9.98	1.57E-15	GO:0003674;GO:0003824;GO:0005488;GO:0005515;GO:0008150;GO:0008152;GO:0016491;GO:0016701;GO:0016702;GO:0043167;GO:0043169;GO:0046872;GO:0051213;GO:0055114	-
7 d	MS.gene02120	hypothetical protein DVH24_022195 [Malus domestica]	RXH86922.1	-10.2	1.99E-34	GO:0000105;GO:0000166;GO:0003674;GO:0003824;GO:0004399;GO:0005488;GO:0005515;GO:0006082;GO:0006520;GO:0006547;GO:0006725;GO:0006807;GO:0008150;GO:0008152;GO:0008270;GO:0008652;GO:0009058;GO:0009072;GO:0009073;GO:0009987;GO:0016053;GO:0016491;GO:0016614;GO:0016616;GO:0018130;GO:0019438;GO:0019752;GO:0034641;GO:0036094;GO:0043167;GO:0043169;GO:0043436;GO:0044237;GO:0044238;GO:0044249;GO:0044281;GO:0044283;GO:0046394;GO:0046483;GO:0046872;GO:0046914;GO:0048037;GO:0050662;GO:0051287;GO:0052803;GO:0055114;GO:0071704;GO:0097159;GO:1901265;GO:1901360;GO:1901362;GO:1901363;GO:1901564;GO:1901566;GO:1901576;GO:1901605;GO:1901607	K00013
7 d	MS.gene99553	MLP-like protein 28 [Medicago truncatula]	XP_013445246.1	-10.23	7.31E-25	GO:0006950;GO:0006952;GO:0008150;GO:0050896	-
7 d	MS.gene61470	early nodulin-93 [Medicago truncatula]	XP_003609265.1	-11.06	9.92E-20	-	-

[1]NR ID is the protein accession number in NCBI non redundant protein database.
[2]log_2FC stands for log Fold Change, where it is log base 2.
[3]FDR is adjusted P value.
