# Supplementary material for: Transcriptome Analysis of Fusarium Root-Rot-Resistant and -Susceptible Alfalfa (Medicago sativa L.) Plants during Plant–Pathogen Interactions
Source: Genes (Basel). 2022 Apr 28;13(5):788. doi: 10.3390/genes13050788 (PMC9140628; doi:10.3390/genes13050788)
Supplement: Supplementary file 1 [file genes-13-00788-s001.zip › Table S8.pdf]

Table S8: Top 30 fungal genes detected in the two lines at four time points.

Gene ID[a]	Chromosome	Start Site	End Site	Direction	Length	Description[b]	TS24 (Read Count)	TS48 (Read Count)	TS72 (Read Count)	TS7d (Read Count)	TR24 (Read Count)	TR48 (Read Count)	TR72 (Read Count)	TR7d (Read Count)
gene-FPRO_13465	NW_022194801.1	1060189	1061907	-	1654	uncharacterized protein	28311	6470	1734	2436	487	2756	640	1382
gene-FPRO_03476	NW_022194791.1	945807	946928	-	3170	uncharacterized protein	24164	4456	1764	2366	375	1855	702	1062
gene-FPRO_00137	NW_022194790.1	6012150	6012748	+	217	related to hydrophobin	2906	702	133	333	72	355	100	159
gene-FPRO_03131	NW_022194791.1	1970471	1972240	+	1141	related to tyrosinase precursor	2972	726	373	621	290	508	442	348
gene-FPRO_00480	NW_022194790.1	4958891	4959874	+	787	hypothetical protein	2629	417	840	3656	1004	631	2700	876
gene-FPRO_14358	NW_022194797.1	1720754	1721072	-	474	hypothetical protein	2149	329	221	424	198	214	524	151
gene-FPRO_02896	NW_022194791.1	2717784	2732433	+	144	non-ribosomal peptide synthetase	1872	286	172	307	136	185	382	120
gene-FPRO_13093	NW_022194801.1	14485	14823	-	1474	uncharacterized protein	2001	330	375	1040	229	320	755	342
gene-FPRO_01381	NW_022194790.1	2152709	2155519	+	549	suppressor protein PSP1	1915	314	705	2732	242	272	2067	640
gene-FPRO_01477	NW_022194790.1	1856542	1858474	-	1358	uncharacterized protein	1789	336	541	1570	371	357	1131	420
gene-FPRO_09277	NW_022194795.1	596972	598739	-	2506	translation elongation factor 1-alpha	1677	366	216	708	249	363	291	432
gene-FPRO_01102	NW_022194790.1	2974752	2975440	-	1772	hypothetical protein	1589	303	397	889	180	281	550	255
gene-FPRO_08409	NW_022194800.1	650553	653315	-	98	uncharacterized protein	1572	156	93	218	111	99	178	92
gene-FPRO_07610	NW_022194796.1	1743823	1746533	+	788	uncharacterized protein	1456	220	280	787	223	233	697	239
gene-FPRO_08028	NW_022194796.1	459302	460417	-	69	uncharacterized protein	1209	163	31	61	29	70	61	36
gene-FPRO_10907	NW_022194794.1	2005805	2006653	+	635	uncharacterized protein	1334	238	200	488	133	182	273	179
gene-FPRO_13469	NW_022194801.1	1076277	1076543	+	301	uncharacterized protein	1155	170	187	399	79	116	240	151
gene-FPRO_00850	NW_022194790.1	3776049	3776377	+	680	uncharacterized protein	1278	186	199	326	144	171	283	130
gene-FPRO_00907	NW_022194790.1	3593313	3614232	+	238	uncharacterized protein	1185	170	153	511	118	133	289	168
gene-FPRO_14475	NW_022194797.1	1983689	1984845	-	625	uncharacterized protein	1248	193	195	644	130	173	370	204
gene-FPRO_00214	NW_022194790.1	5803824	5809166	+	128	uncharacterized protein	1151	158	83	175	35	77	68	65
gene-FPRO_04107	NW_022194803.1	243779	244759	-	347	opsin-like protein	1186	172	145	567	116	135	341	166
gene-FPRO_00496	NW_022194790.1	4897595	4901411	+	1021	plasma membrane ATPase	1269	267	163	566	212	244	244	196
gene-FPRO_01890	NW_022194790.1	673884	675530	+	1387	probable subtilisin-like serine protease	1198	246	428	1108	227	313	928	352
gene-FPRO_05070	NW_022194793.1	2782832	2783744	-	380	-	1175	182	100	150	121	122	191	104
gene-FPRO_15107	NW_022194798.1	1798691	1799779	+	454	uncharacterized protein	1089	157	265	660	130	152	347	104
gene-FPRO_09570	NW_022194795.1	1519989	1520921	-	796	probable transcription initiation factor TFIID	1120	173	195	757	156	178	464	230
gene-FPRO_13354	NW_022194801.1	773387	775018	-	441	RAN protein kinase	1091	186	297	903	126	156	500	187
gene-FPRO_09334	NW_022194795.1	760750	762126	-	559	uncharacterized protein	991	180	286	792	188	242	642	322
gene-FPRO_03100	NW_022194791.1	2070524	2071605	+	1374	uncharacterized protein	1046	213	283	772	238	212	577	207

[a]Fungal genes are based on *F.proliferatum* ET1 NCBI Id.

[b]Descriptions of fungal genes are based on the NCBI database.
