# Supplementary figures and images for: Transcriptome Analysis of Fusarium Root-Rot-Resistant and -Susceptible Alfalfa (Medicago sativa L.) Plants during Plant–Pathogen Interactions
Source: Genes (Basel). 2022 Apr 28;13(5):788. doi: 10.3390/genes13050788 (PMC9140628; doi:10.3390/genes13050788)

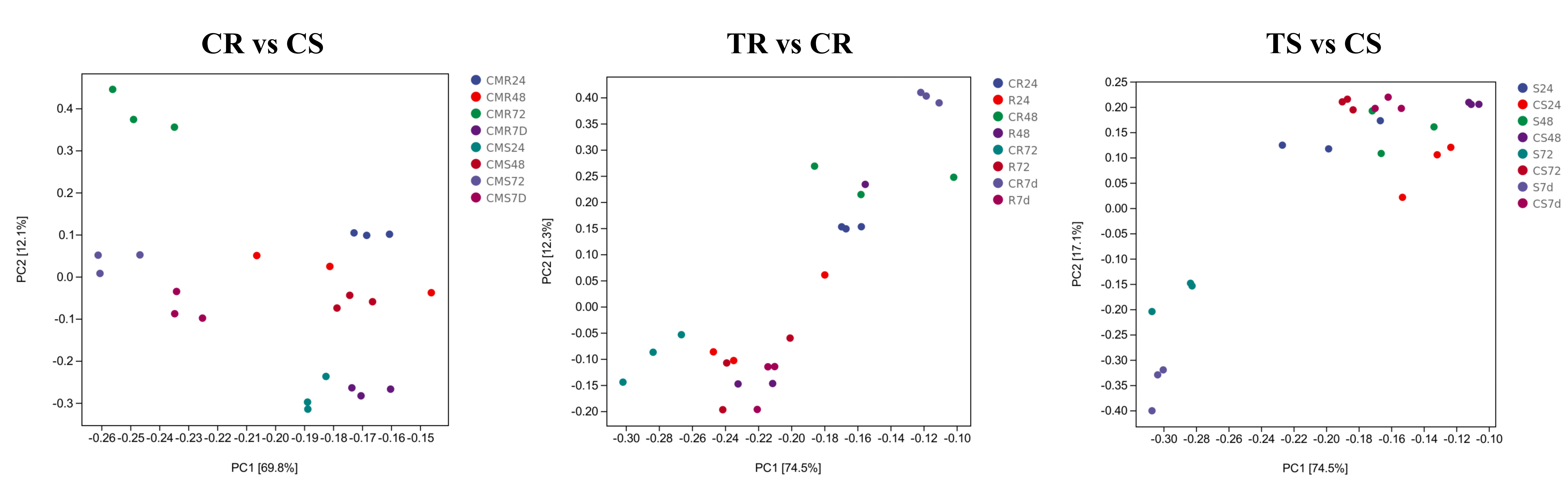

Supplement: Supplementary file 1 [file genes-13-00788-s001.zip › Figure S1.tif]

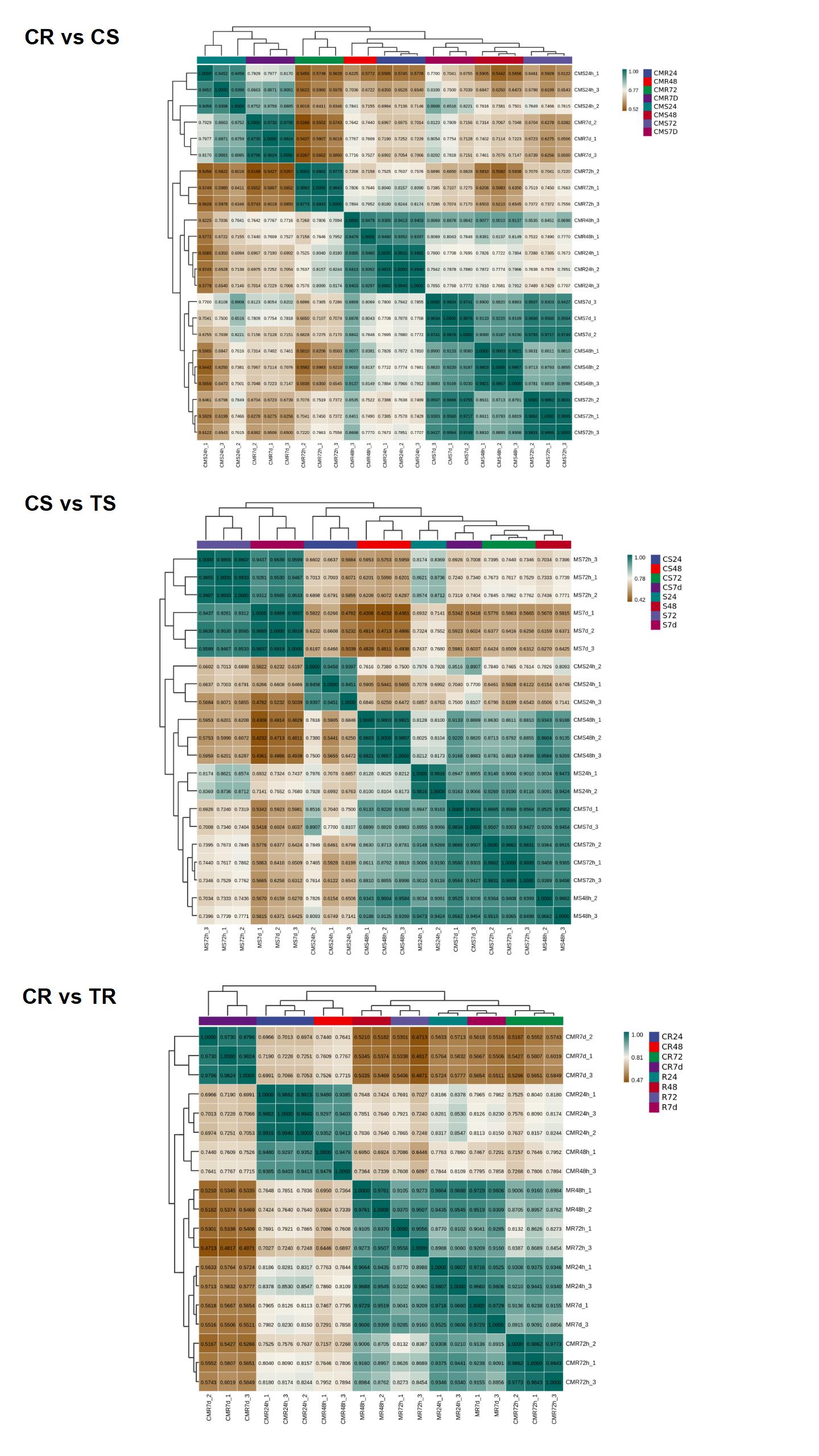

Supplement: Supplementary file 1 [file genes-13-00788-s001.zip › Figure S2.tif]

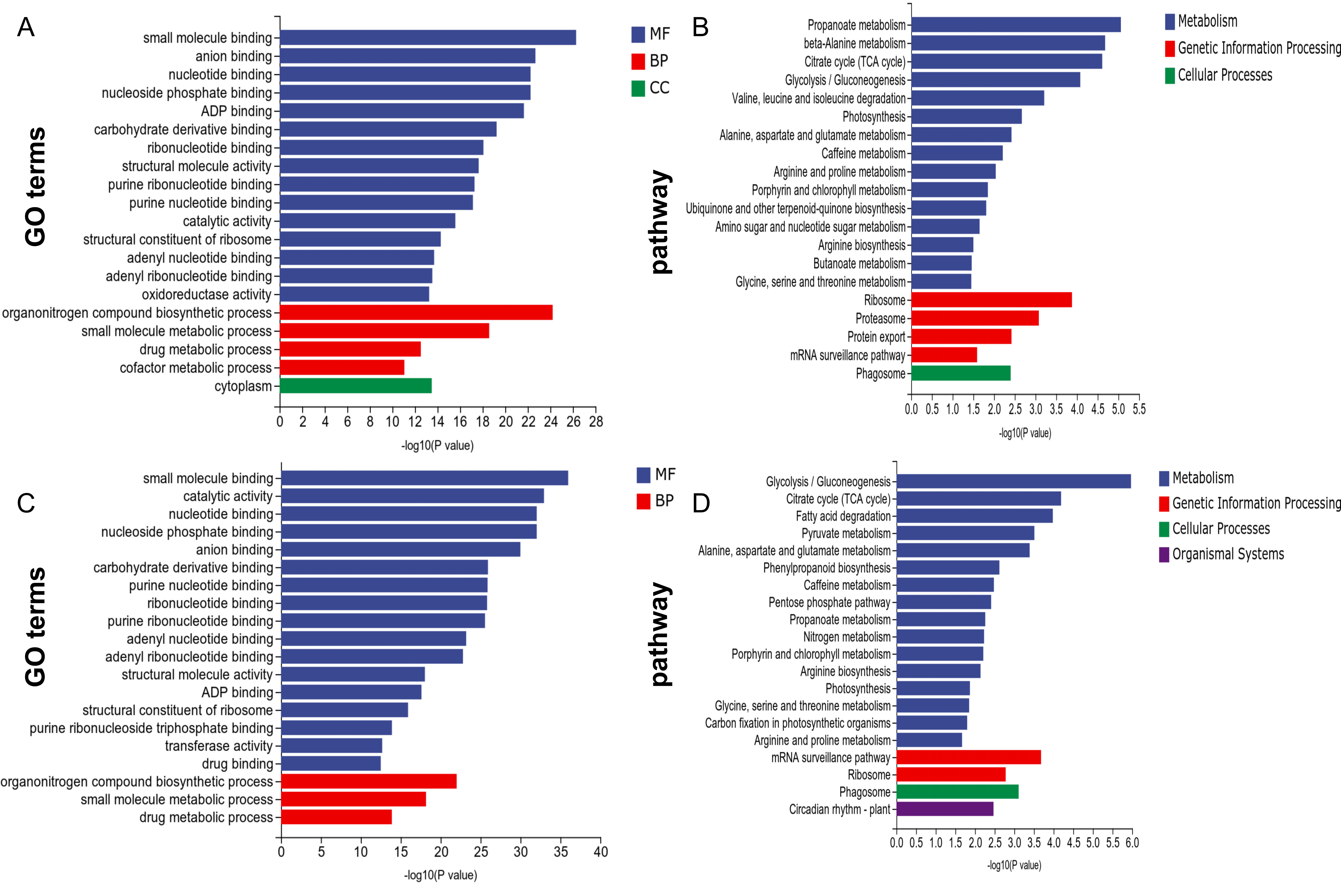

Supplement: Supplementary file 1 [file genes-13-00788-s001.zip › Figure S4.tif]

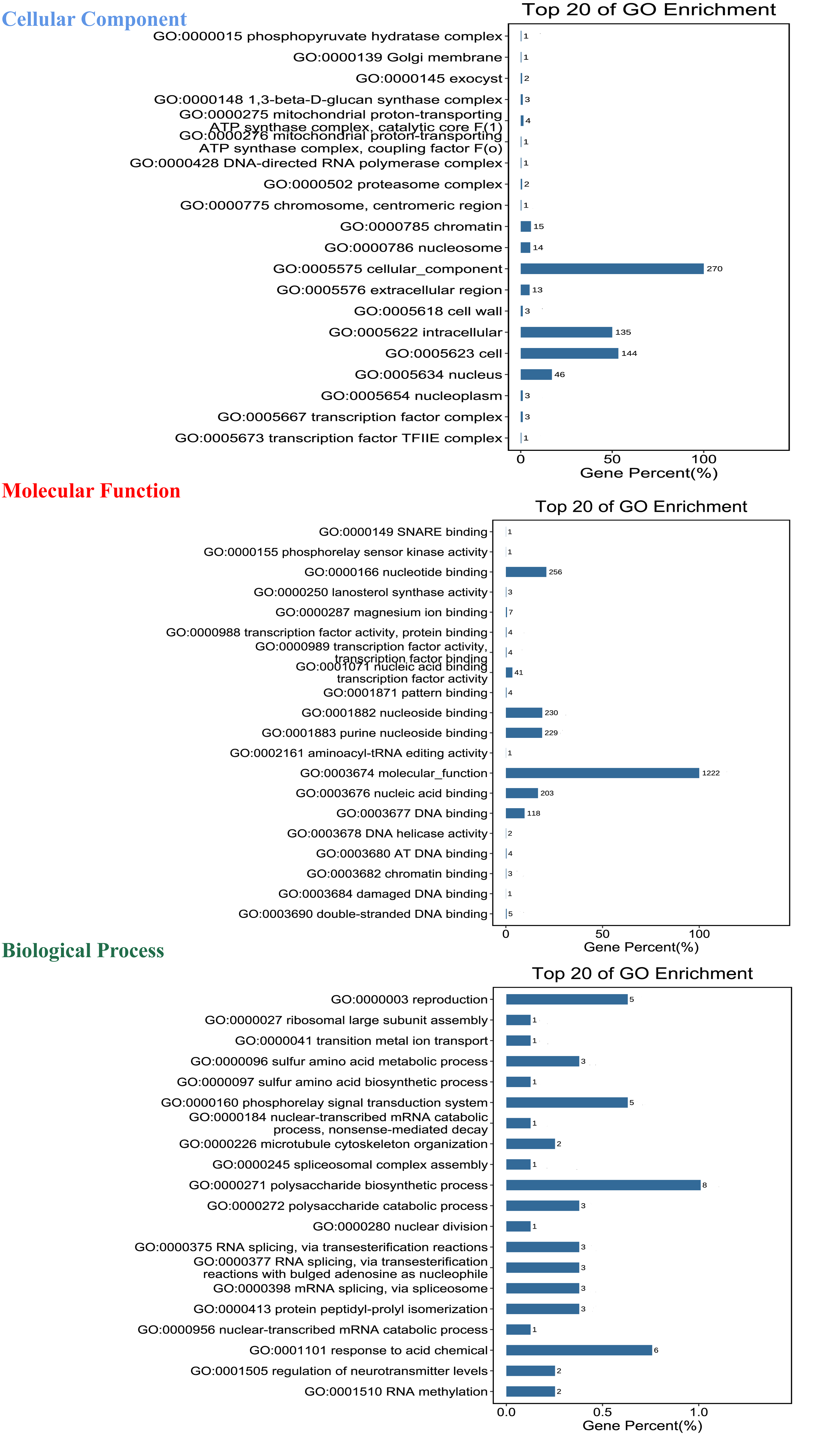

Supplement: Supplementary file 1 [file genes-13-00788-s001.zip › Figure S5.tif]

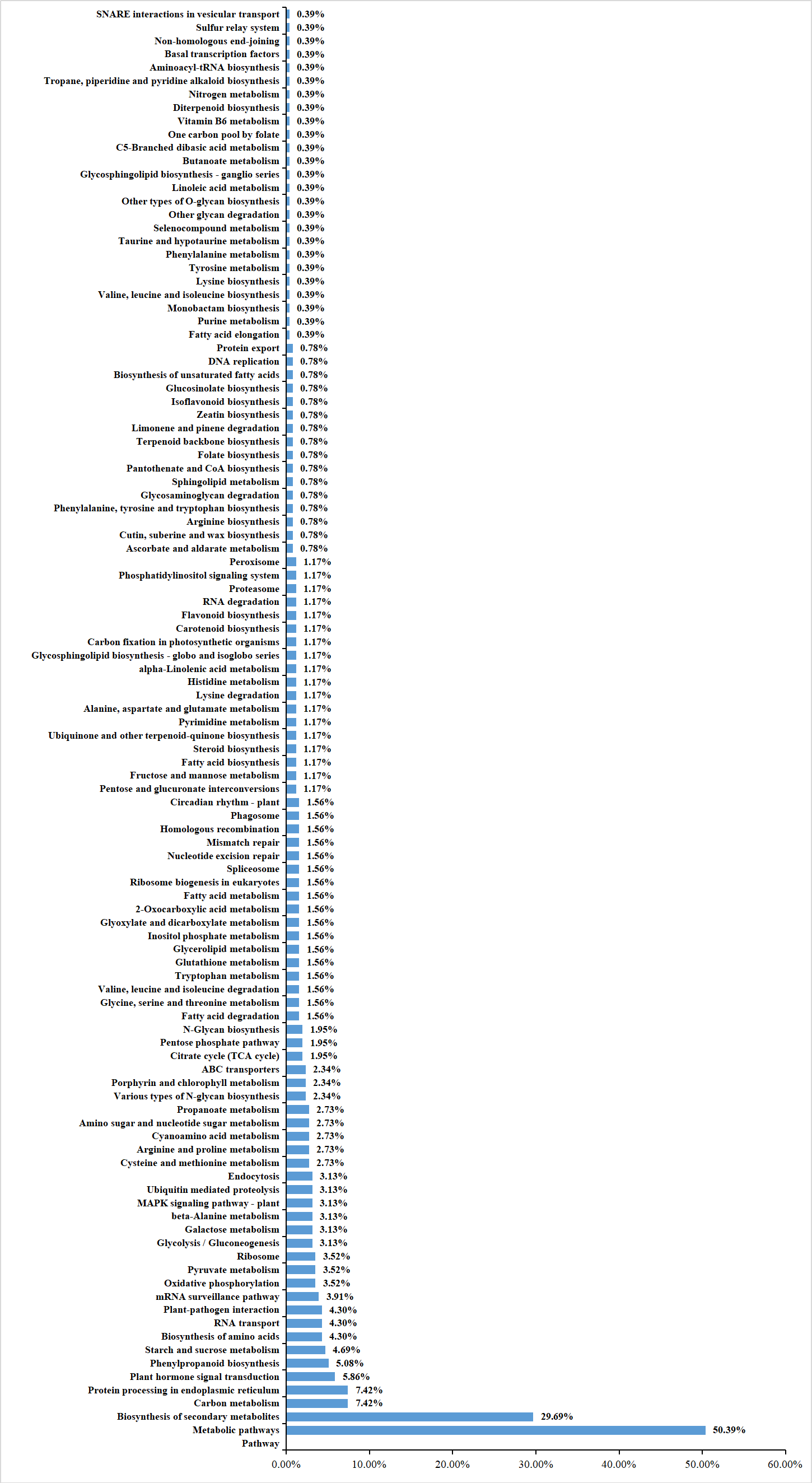

Supplement: Supplementary file 1 [file genes-13-00788-s001.zip › Figure S6.tif]

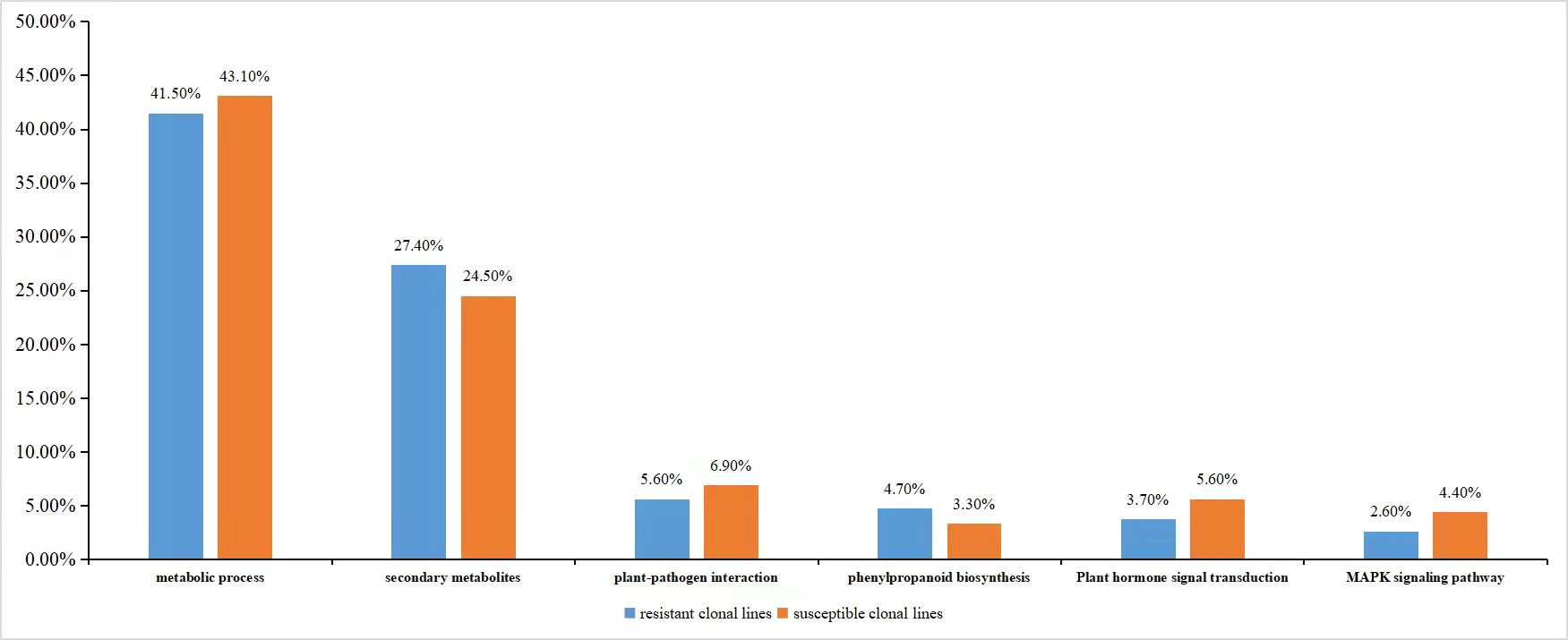

Supplement: Supplementary file 1 [file genes-13-00788-s001.zip › Figure S7.tif]
